# Activation of an actin signaling pathway in pre-malignant mammary epithelial cells by P-cadherin is essential for transformation

**DOI:** 10.1242/dmm.049652

**Published:** 2023-02-21

**Authors:** Lídia Faria, Sara Canato, Tito T. Jesus, Margarida Gonçalves, Patrícia S. Guerreiro, Carla S. Lopes, Isabel Meireles, Eurico Morais-de-Sá, Joana Paredes, Florence Janody

**Affiliations:** ^1^ i3S, Instituto de Investigação e Inovação em Saúde, Universidade do Porto, Rua Alfredo Allen 208, 4200-135 Porto, Portugal; ^2^ Instituto de Patologia e Imunologia Molecular da Universidade do Porto (Ipatimup), Rua Júlio Amaral de Carvalho, n 45, 4200-135 Porto, Portugal; ^3^ Master Programme in Oncology, School of Medicine and Biomedical Sciences, University of Porto (ICBAS-UP), Rua Jorge Viterbo Ferreira 228, 4050-513 Porto, Portugal; ^4^ Physiology and Cancer Program, Champalimaud Foundation, Avenida de Brasília, 1400-038 Lisboa, Portugal; ^5^ Instituto de Biologia Molecular e Celular (IBMC), Universidade do Porto, Rua Alfredo Allen 208, 4200-135 Porto, Portugal; ^6^ Vector B2B - Drug Developing - Associação Para Investigação em Biotecnologia, Av. Prof. Egas Moniz, Edifício Egas Moniz, 1649-028 Lisboa, Portugal; ^7^ FMUP, Medical Faculty of University of Porto, Alameda Prof. Hernâni Monteiro, 4200-319 Porto, Portugal; ^8^ Instituto Gulbenkian de Ciência, Rua da Quinta Grande 6, P-2780-156 Oeiras, Portugal

**Keywords:** Actin cytoskeleton, *Drosophila*, Human mammary epithelial cells, MRTF-A–SRF signaling, P-cadherin, Pre-malignant lesions

## Abstract

Alterations in the expression or function of cell adhesion molecules have been implicated in all steps of tumor progression. Among those, P-cadherin is highly enriched in basal-like breast carcinomas, playing a central role in cancer cell self-renewal, collective cell migration and invasion. To establish a clinically relevant platform for functional exploration of P-cadherin effectors *in vivo*, we generated a humanized P-cadherin *Drosophila* model. We report that actin nucleators, Mrtf and Srf, are main P-cadherin effectors in fly. We validated these findings in a human mammary epithelial cell line with conditional activation of the *SRC* oncogene. We show that, prior to promoting malignant phenotypes, SRC induces a transient increase in P-cadherin expression, which correlates with MRTF-A accumulation, its nuclear translocation and the upregulation of SRF target genes. Moreover, knocking down P-cadherin, or preventing F-actin polymerization, impairs SRF transcriptional activity. Furthermore, blocking MRTF-A nuclear translocation hampers proliferation, self-renewal and invasion. Thus, in addition to sustaining malignant phenotypes, P-cadherin can also play a major role in the early stages of breast carcinogenesis by promoting a transient boost of MRTF-A–SRF signaling through actin regulation.

## Teaser

**Summary:** We provide evidence that a transient boost of actin–MRTF-A–SRF signaling activity by the cell–cell adhesion molecule P-cadherin could play a major role in pre-malignant disease development and progression.

## INTRODUCTION

Cell adhesion molecules (CAMs) are transmembrane receptor proteins widely expressed in epithelial tissues. Although their primary role is to maintain cell–cell contact and attachment to the extracellular matrix, they also integrate extracellular cues within cell-intrinsic signaling, affecting cytoskeleton organization, intracellular responses and gene expression, and consequently cellular functions, such as cell growth, survival and invasion. It is therefore not surprising that alterations in their expression and/or activity influence malignant transformation, being considered potential targets for cancer therapy (Makrilia et al., 2009). Yet, the mechanisms by which many of these CAMs impact cancer malignancy remain unclear.

Aberrant expression of the classical cell–cell adhesion molecule P-cadherin (P-cad; encoded by the *CDH3* gene, referred to here as *P-cad*) has been associated with aggressive tumor behavior in breast, gastric, prostate, pancreatic, bladder and colorectal carcinomas, among others (Vieira and Paredes, 2015). In breast cancer, P-cad is overexpressed in a molecular subset of highly aggressive basal-like triple-negative carcinomas, being significantly associated with worse disease-free and overall patient survival (Paredes et al., 2005). P-cad promotes cell motility, collective cell migration and invasion capacity in breast cancer cells *in vitro* (Ribeiro et al., 2010). In addition, P-cad instructs cancer cells to acquire stem-like cell properties, thus contributing to the survival of aggressive breast cancer cells, and induces tumorigenic and metastatic capacity in *in vivo* breast cancer models (Paredes et al., 2004; Ribeiro et al., 2010, 2013; Vieira et al., 2012, 2014). Molecularly, P-cad activates α6β4 integrins and the non-receptor tyrosine kinase SRC, and interferes with E-cadherin (E-cad; encoded by the *CDH1* gene) function (Ribeiro et al., 2013, 2018; Vieira et al., 2014). In addition, P-cad promotes GTPase-mediated signal transduction and affects the actin cytoskeleton (Taniuchi et al., 2005; Ribeiro et al., 2013, 2016). Yet, how the actin cytoskeleton dynamics integrates into the P-cad-dependent signaling network remains to be established. Clarifying this network is fundamental to uncover potential therapeutic targets that could be used to counteract P-cad-mediated tumorigenic capacity.

Filamentous actin (F-actin) is assembled from monomeric actin subunits (G-actin). Polymerization occurs predominantly by extension of the fast-growing barbed ends of filaments, largely facilitated by the activity of diverse actin nucleators. Among those, the Arp2/3 complex, formed by seven subunits (Arp2, Arp3 and Arpc1-5), catalyzes polymerization of new ‘daughter’ filaments from the side of existing filaments to form branched networks. In contrast, formins generate the formation of linear, unbranched actin filaments, while Spire, a tandem-monomer-binding nucleator, recruits actin monomers to form polymerization seeds (Chesarone and Goode, 2009). In addition to controlling key cellular processes that include the generation and maintenance of cell morphology and polarity, endocytosis, intracellular trafficking, contractility and cell division, the actin cytoskeleton is a major regulator of signal transduction pathways (Moujaber and Stochaj, 2020). One of those is the myocardin-related transcription factor A (MRTF-A)–serum response factor (SRF) signaling pathway. MRTF-A contains a conserved N-terminus RPEL (arginine-proline-glutamine-leucine consensus sequence-containing) domain that includes three actin-binding motifs (RPEL1, RPEL2, RPEL3), overlapping with an extended bipartite nuclear localization signal (NLS) (Pawłowski et al., 2010; Mouilleron et al., 2011). In the absence of stimulation, MRTF-A localizes in the cytoplasm due to its association with G-actin through the RPEL motifs (Mouilleron et al., 2011). This hinders the NLS of MRTF-A, preventing its recognition by the importin-α/β heterodimer and, thus, blocking its nuclear import (Pawłowski et al., 2010). Increased F-actin polymerization depletes the pool of G-actin, which, consequently, dissociates from MRTF-A, allowing the access of import factors to the NLS to promote MRTF-A nuclear translocation, where it binds to SRF and activates the expression of target genes (Vartiainen et al., 2007; Pawłowski et al., 2010). In turn, MRTF-A/SRF affect the actin cytoskeleton by regulating the expression of actin and of proteins controlling F-actin nucleation and organization, explaining why the MRTF-A–SRF pathway plays a central role in the mobility of normal and cancer cells (Gau and Roy, 2018). Indeed, increasing evidence indicates that MRTF-A has oncogenic properties, promoting proliferation, epithelial–mesenchymal transition (EMT), stemness abilities and metastasis (Seifert and Posern, 2017; Gasparics and Sebe, 2018; Gau and Roy, 2018).

Therapeutic approaches that would counteract the tumorigenic capacity of P-cad depend on a detailed understanding of its downstream signaling network. To this end, we generated a fly model with conditional expression of human P-cad, as the large arsenal of genetic tools in *Drosophila*, the rapid generation time and the high levels of conservation in genes encoding for cancer signaling pathways allow powerful studies of underlying mechanisms (Villegas et al., 2019). We show that our humanized P-cad fly model is a clinically relevant platform for functional exploration of P-cad effectors *in vivo*, and we identified *Drosophila* actin nucleators, Mrtf and Srf, as suppressors of the P-cad-expressing wing phenotype. We validated the role of the P-cad-dependent actin–MRTF-A–SRF signaling pathway in a human breast epithelial cell line, which recapitulates molecular events taking place during the transition from a normal to a malignant state. We provide evidence that P-cad plays a central role in the very early stages of breast carcinogenesis by promoting a transient boost of actin–MRTF-A–SRF signaling pathway activity, essential for the acquisition of pre-malignant and malignant phenotypes.

## RESULTS

### Human P-cad is functional in *Drosophila* epithelia

**Fig. 1.**
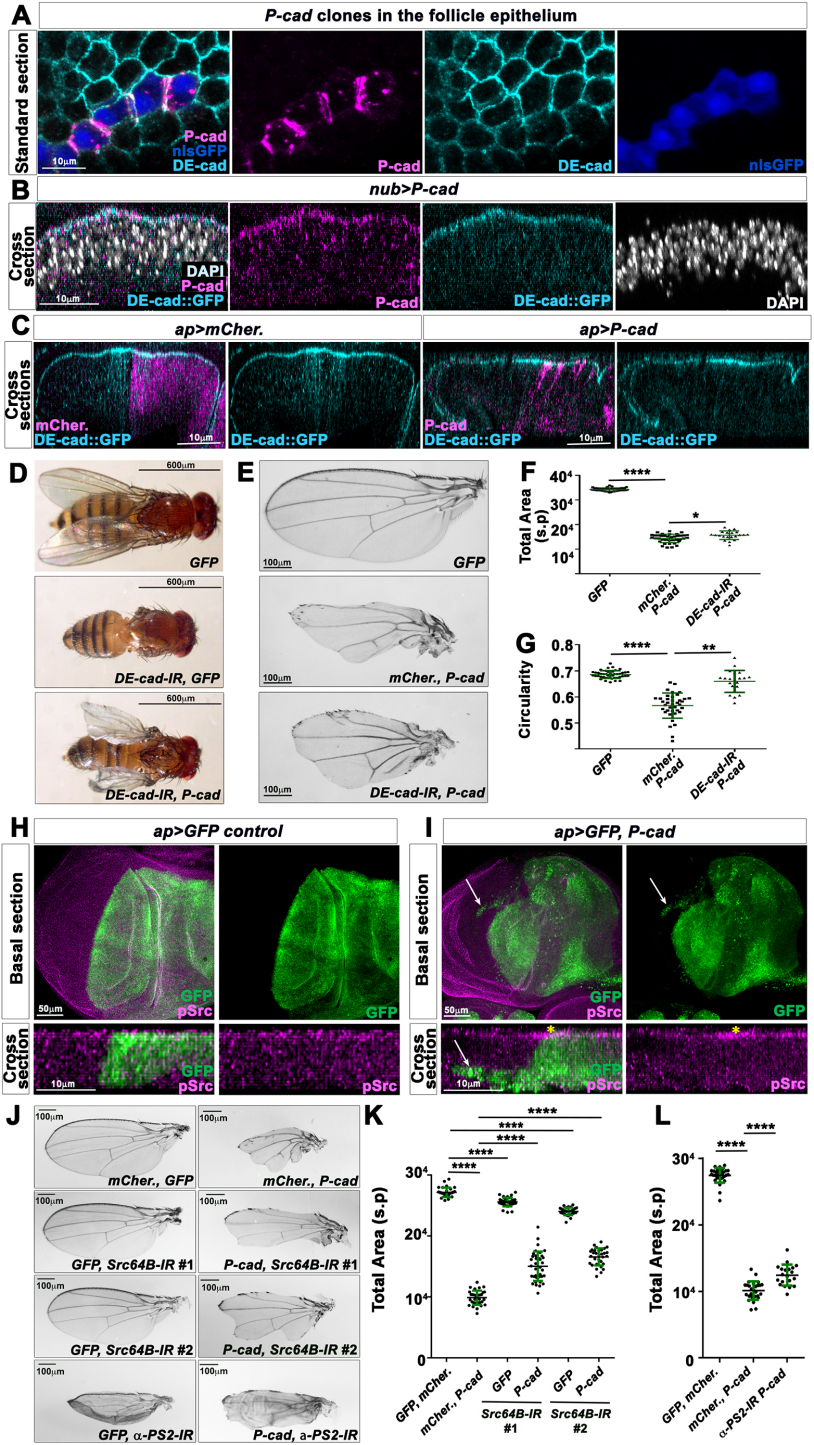
**Human P-cadherin (P-cad) colocalizes with *Drosophila* E-cadherin (DE-cad) apically in *Drosophila* epithelia and affects wing development through DE-cad, Src64B and αPS2 integrin.** (A) Standard confocal sections of P-cad-expressing clones marked with nuclear GFP (nlsGFP; blue) in the follicle epithelium of a stage 6 egg chamber, stained with anti-DE-cad (cyan) and anti-P-cad (magenta). Scale bar: 10 µm. (B) Cross-section of third-instar wing imaginal discs carrying a GFP knock-in into the DE-cad locus (cyan) and expressing UAS-*P-cad* under *nub-*Gal4 control, stained with anti-P-cad (magenta) and DAPI (white). Scale bar: 10 µm. (C) Cross-section of third-instar wing imaginal discs carrying a GFP knock-in into the DE-cad locus (cyan), expressing UAS-*mCherry* (*mCher.*) (magenta) or UAS-*P-cad* under *ap-*Gal4 control, stained with anti-P-cad (magenta). Scale bar: 10 µm. Staining in A-C was performed twice. (D,E) Adult flies (D) or wings (E) in which *nub-*Gal4 drives the indicated UAS-constructs. Scale bars: 600 µm (D) and 100 µm (E). (F,G) Quantifications from one biological replicate performed in parallel for total area (F) and circularity (G) for wings in which *nub-*Gal4 drives UAS-*CD8-GFP* (*n*=31), UAS-*mCherry* and UAS-*P-cad* (*n*=29) or UAS-*DE-cad-IR* and UAS-*P-cad* (*n*=20). (H,I) Standard confocal sections (top row) or cross-sections (bottom row) of third-instar wing imaginal discs expressing UAS*-mCD8-GFP* (green) (H) or UAS-*mCD8-GFP* (green) and UAS-*P-cad* (I) using the *ap*-Gal4 driver, stained with anti-phosphorylated Src (pSrc) (magenta). Scale bars: 50 µm (top row) and 10 µm (bottom row). White arrows indicate cells migrating in the ventral compartment. Yellow asterisks indicate the increase in apical pSrc levels in the dorsal compartment expressing P-cad. Staining in H and I was performed once. (J) Adult wings in which *nub-*Gal4 drives the indicated UAS constructs. Scale bars: 100 µm. (K,L) Quantifications of the total area for wings in which *nub-*Gal4 drives UAS-*CD8-GFP* and UAS-*mCherry* (*n*=29), or UAS-*mCherry* and UAS-*P-cad* (*n*=32), or UAS-*mCD8-GFP* and UAS-*Src64B-IR* #1 (*n*=40), or UAS-*P-cad* and UAS-*Src64B-IR* #1 (*n*=36), or UAS-*mCD8-GFP* and UAS-*Src64B-IR* #2 (*n*=30), or UAS-*P-cad* and UAS-*Src64B-IR* #2 (*n*=31) (K); or UAS-*CD8-GFP* and UAS-*mCherry* (*n*=30), or UAS-*mCherry* and UAS-*P-cad* (*n*=27), or UAS-*P-cad* and UAS-*α-PS2-IR* (*n*=20) (L). s.p., square pixels. Quantifications of control *nub>mCherry, GFP* and *nub>mCherry, P-cad* are from four or six independent crosses, respectively. Other quantifications are from one biological replicate. Error bars indicate s.d.; *P<0.05; **P<0.01; ****P<0.0001. Statistical significance was calculated using one-way ANOVA with Tukey's multiple comparison.

Basal-like breast carcinomas that overexpress P-cad also maintain high levels of E-cad (Paredes et al., 2005). *Drosophila* E-cad (DE-cad; also known as Shotgun) plays an analogous role to vertebrate E-cad in epithelial polarity (Oda and Takeichi, 2011). Thus, to identify P-cad effectors relevant to its functional effect in cancer, we decided to establish a *Drosophila* model that would mirror carcinomas expressing both DE-cad endogenously and human P-cad, by generating transgenic fly strains carrying human P-cad inducible with the Gal4-UAS system. This allowed us to control P-cad expression both temporally and spatially during fly development. To validate the humanized P-cad fly model, we searched for evidence that some P-cad-induced functional effects were recapitulated in fly epithelia. When mosaic expression was induced in the follicular epithelium, P-cad colocalized with DE-cad apically. Strikingly, P-cad was found to accumulate specifically at the cellular membranes juxtaposed to other P-cad-expressing cells (Fig. 1A), suggesting that P-cad establishes *trans*-junctional homophilic interactions in fly epithelia. Cross-sections through the wing disc epithelium showed that P-cad expressed with the *nubbin-*Gal4 (*nub-*Gal4) driver also accumulated apically with DE-cad (Fig. 1B). P-cad does not appear to affect DE-cad levels or localization, as no apparent difference in DE-cad levels in the follicle epithelium was observed between *P-cad*-expressing cells and cells that did not express *P-cad* (Fig. 1A). Moreover, in wing disc cells expressing *P-cad* in the dorsal compartment using the *apterous*-Gal4 (*ap-*Gal4) driver, DE-cad levels and localization were not affected when compared to ventral cells used as control (Fig. 1C). Thus, like in human cells (Ribeiro et al., 2013), P-cad colocalizes with DE-cad in *Drosophila* epithelia.

Because P-cad has also been shown to compensate for the loss of E-cad in human cells (Ribeiro et al., 2013; Bazellières et al., 2015), we asked whether human P-cad could substitute for DE-cad function. Indeed, although flies expressing a RNA interference (RNAi) construct against DE-cad in the wing epithelial primordium with *nub*-Gal4 failed to form wings, expressing P-cad in those animals partially restored wing development (Fig. 1D). Conversely, human E-cad is required for P-cad-induced tumor growth (Ribeiro et al., 2013). We therefore asked whether reducing DE-cad function suppressed the small, notched wing phenotype induced by P-cad expression. Actually, knocking down DE-cad in these wings significantly restored their growth and circularity (Fig. 1E-G). Thus, in fly wing epithelia, P-cad appears to show similar interactions with DE-cad to those reported with human E-cad in cancer cells (Ribeiro et al., 2013; Bazellières et al., 2015).

In breast cancer cells, P-cad is also known to promote cell motility, collective cell migration and invasive capacities by potentiating the activation of the SRC proto-oncogene and by signaling through SRC and α6β4 integrins (Ribeiro et al., 2010, 2018; Vieira et al., 2014). Thus, we tested whether *P-cad*-expressing wing disc cells behave likewise. Whereas dorsal wing disc cells expressing GFP under *ap-*Gal4 maintained a straight boundary with GFP-negative ventral cells (Fig. 1H), those expressing *P-cad* with GFP suffered basal cell extrusion and migrated within the ventral compartment (see arrows in Fig. 1I). In addition, P-cad appeared to potentiate the levels of phosphorylated Src (pSrc) apically compared to the ventral compartment, which was used as control (Fig. 1I). We then tested whether P-cad would signal through *Drosophila* Src and integrins. Expressing two independent UAS-RNAi constructs against *Drosophila* Src64B had only marginal effects on wing size. However, RNAi could markedly restore the growth defects of *nub>P-cad*-expressing wings (Fig. 1J,K). Moreover, knocking down the integrin *αPS2* (also known as *inflated*) in *P-cad*-expressing wings also significantly restored wing growth, despite the penetrant inflated phenotype induced by reducing αPS2 function alone (Fig. 1J,L).

The reduced size of P-cad-expressing wings could be the consequence of defects in cell growth, proliferation or survival. Wing disc primordia expressing *P-cad* and *GFP* with *nub-*Gal4 only displayed low levels of activated Caspase 3-positive staining (Fig. S1A), suggesting that the reduced size of P-cad-expressing wings is not only a consequence of tissue loss by apoptosis. To test whether blocking Caspases could rescue the P-cad-expressing adult wing phenotype, we expressed the baculovirus *p35*, which has been proposed to restrict wing growth through its inhibitory effect on Caspases, but independently of apoptosis (Ding et al., 2016; Shinoda et al., 2019). Expressing *p35* in *nub>GFP* wing imaginal discs reduced adult wing size, as previously reported (Ding et al., 2016; Shinoda et al., 2019), and did not restore the size of *nub>P-cad* adult wings. Instead, these wings showed a worse phenotype (Fig. S1B). These observations suggest an additive effect between p35 and P-cad on wing growth, involving impairment of cell growth and proliferation by p35. Yet, these observations do not allow us to conclude on whether P-cad affects tissue growth by preventing cell growth, proliferation or survival. To test the role of cell death downstream of P-cad, we therefore analyzed whether overexpressing *Death-associated inhibitor of apoptosis 1* (*Diap1*) suppressed the P-cad-expressing wing phenotype, as Diap1 inhibits apoptotic Caspase activity (Meier et al., 2000). Overexpressing *Diap1* could significantly restore the growth defect of *nub>P-cad*-expressing wings and enhanced the growth of wild-type wings expressing *GFP* (Fig. S1C). Thus, tissue loss by apoptosis could contribute to the reduced size of P-cad-expressing wings. Yet, *nub>P-cad, Diap1* wings remained small compared to control *nub>GFP* wings (Fig. S1C), suggesting that P-cad also affects cell proliferation. Accordingly, the ratio of phosphorylated Histone 3 (pH3) signals between the dorsal blade compartment and the total blade domain was reduced in wing cells expressing *P-cad* and *GFP* under *ap-*Gal4 control, compared to that in wing cells expressing *GFP* only (Fig. S2). Thus, the reduced size of P-cad-expressing wings results from a decreased proliferative ability of wing blade cells, associated with a propensity to undergo apoptosis.

Expressing P-cad in the eye-antenna primordia, using the *eyeless-*Gal4 (*ey-*Gal4) driver, also markedly affected the development of the fly head (Fig. S3A). However, when expressed in cells posterior to the morphogenetic furrow using *Glass Multimer Reporter*-Gal4 (*GMR*-Gal4), P-cad-expressing adult eyes displayed only a marginal rough eye phenotype, unlike adult eyes expressing *Rho1* used as positive control (Fig. S3B,C). Moreover, quantification of the eye perimeter indicated that *GMR>P-cad*-expressing eyes were enlarged, compared to those of *GMR>mCherry* control (Fig. S3D). These observations suggest that the consequences of overexpressing P-cad are time and/or tissue specific. Accordingly, in human models, the functional effects of P-cad depend on the cellular and tissue context (Vieira and Paredes, 2015).

Taking these results together, we conclude that the consequences of expressing human P-cad in the fly wing epithelium are similar to those of overexpressing P-cad in breast cancer cells (Ribeiro et al., 2013, 2018; Vieira et al., 2014). Thus, the P-cad-dependent wing phenotype is suitable for the identification of P-cad effectors relevant to the acquisition of a tumorigenic phenotype.

### P-cad may potentiate the activity of the Mrtf–Srf signaling pathway

**Fig. 2.**
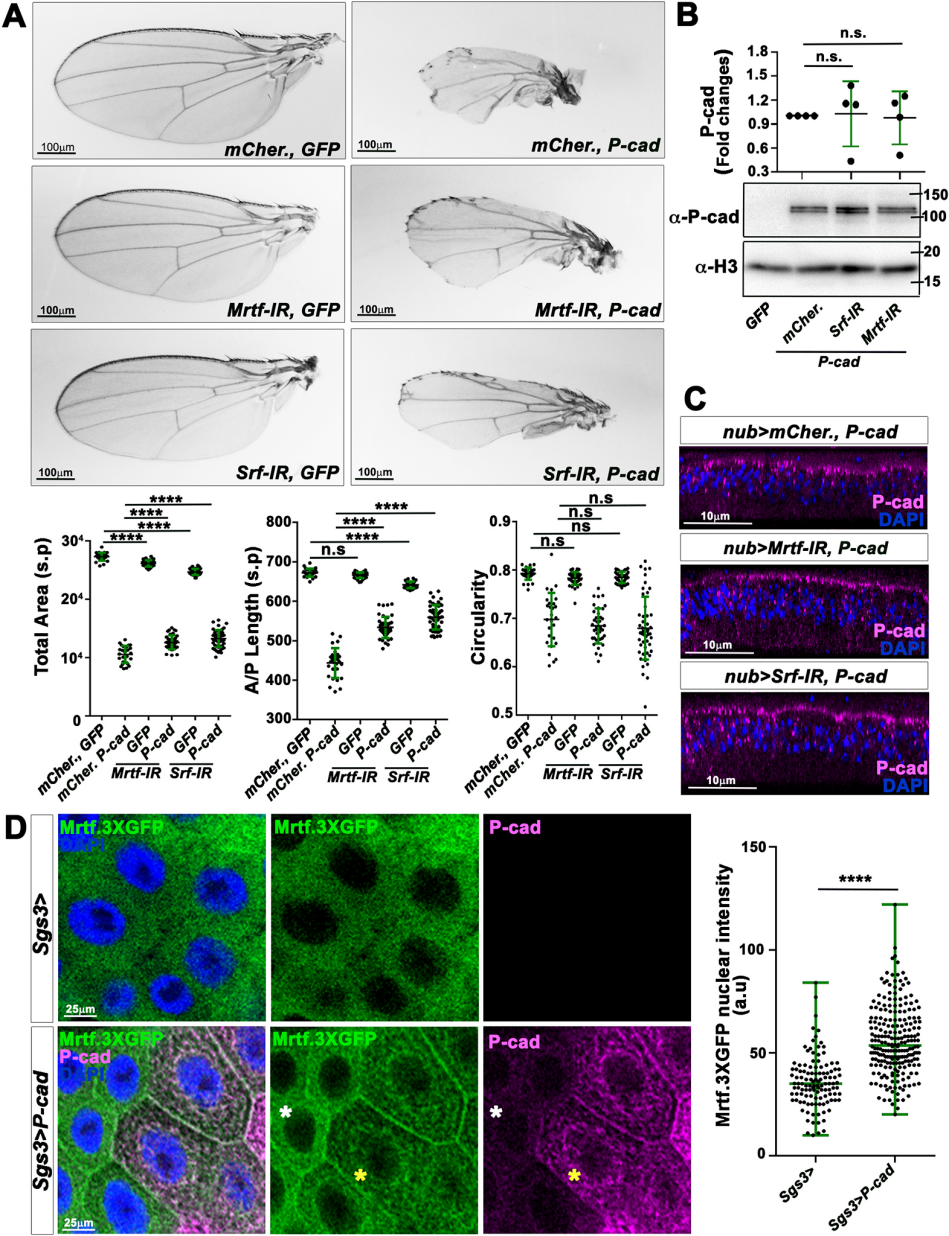
**P-cad might signal through the Mrtf–Srf signaling pathway in the *Drosophila* wing.** (A) (Top) Adult wings in which *nub-*Gal4 drives the indicated UAS constructs*.* Scale bars: 100 µm. (Bottom) Quantifications of the total area (left), anterior–posterior length (middle) or circularity (right) of adult wings in which *nub-*Gal4 drives UAS-*CD8-GFP* and UAS-*mCherry* (*n*=30), or UAS-*mCherry* and UAS-*P-cad* (*n*=27), or UAS-*mCD8-GFP* and UAS-*Srf-IR* (*n*=41), or UAS-*P-cad* and UAS-*Srf-IR* (*n*=39), or UAS-*mCD8-GFP* and UAS-*Mrtf-IR* (*n*=38), or UAS-*P-cad* and UAS-*Mrtf-IR* (*n*=49). Quantifications of control *nub>mCherry, GFP* and *nub>mCherry, P-cad* are from four or six independent crosses, respectively. Other quantifications are from one biological replicate. (B) (Bottom) Western blots on protein extracts from wing imaginal discs expressing the indicated constructs under *nub-*Gal4, blotted with anti-P-cad, with anti-Histone 3 (α-H3) used as loading control. (Top) Quantification from four biological replicates of the ratio of P-cad levels normalized to α-H3 for the genotypes indicated. (C) Cross-sections of third-instar wing imaginal discs expressing the indicated constructs under *nub-*Gal4 control and stained with anti-P-cad (magenta) and DAPI (blue). Scale bars: 10 µm. Staining was performed once. (D) (Left) Standard confocal sections of third-instar salivary glands, expressing *tub*-*Mrtf.3XGFP* (green) or *tub-Mrtf.3XGFP* and UAS-*P-cad* under *Sgs3-*Gal4 control, stained with anti-P-cad (magenta) and DAPI (blue). Yellow and white asterisks indicate cells expressing higher and lower levels of P-cad, respectively. Scale bars: 25 µm. (Right) Quantification from three biological replicates of the nuclear intensity signals of Mrtf.3XGFP for salivary glands in which *Sgs3-*Gal4 drives Tub-*Mrtf.3XGFP* (*n*=117) or Tub*-Mrtf.3XGFP* and UAS-*P-cad* (*n*=227). a.u., arbitrary units. Error bars indicate s.d.; n.s., non-significant; ****P<0.0001. Statistical significance was calculated using one-way ANOVA with Tukey's multiple comparison (A,B) or unpaired two-tailed Student's *t*-test (D).

To identify P-cad effectors, we searched for signaling pathways suppressing the *nub>P-cad* adult wing phenotype when knocked down. We found that adult wings expressing RNAi constructs against *Drosophila* Mrtf (*Mrtf-IR*) or Srf (*Srf-IR*) together with *GFP* under *nub-*Gal4 control were slightly smaller than control wings expressing *GFP* and *mCherry*, but did not display circularity defects. However, although RNAi did not rescue the circularity defect of *nub>P-cad*-expressing wings, in both cases, it could significantly restore the anterior–posterior (A/P) length and total area of these wings (Fig. 2A). The Mrtf–Srf signaling pathway is unlikely to be required to stabilize or localize P-cad, as P-cad levels were not significantly different in wing discs expressing *P-cad* and knocked down for *Mrtf* or *Srf* compared to those expressing *P-cad* and *mCherry* (Fig. 2B; Fig. S4A). Moreover, cross-sections through wing discs epithelia showed that P-cad localization was not altered by reducing Mrtf or Srf function (Fig. 2C). To confirm that P-cad enhances the activity of the Mrtf–Srf signaling pathway, we tested whether P-cad was able to induce the nuclear translocation of a fusion between Mrtf and GFP (Mrtf.3XGFP), which was expressed using the ubiquitous *tubulin* promoter. We used the salivary gland of third-instar larvae, as its monolayer tubular epithelium is composed of larger cells than those of the wing disc epithelium. When driven with the salivary gland-specific driver *Sgs3*-Gal4, the levels of P-cad were very unequal between cells (Fig. 2D). Mrtf.3XGFP localized mainly in the cytoplasm in cells expressing low P-cad levels (white asterisks in Fig. 2D) or in control cells carrying *Sgs3-*Gal4 only. In contrast, in cells with high P-cad levels, Mrtf.3XGFP levels were reduced in the cytoplasm and accumulated faintly in the nucleus (yellow asterisks in Fig. 2D). Quantification of the Mrtf.3XGFP intensity signal indicated that Mrtf.3XGFP accumulated significantly in the nucleus in P-cad-expressing cells (Fig. 2D). Thus, P-cad triggers the nuclear translocation of Mrtf.3XGFP. These observations are consistent with a role for P-cad in potentiating the Mrtf–Srf signaling pathway. Accordingly, knocking down *MESK2* (*MESK2-IR*) or *Deterin* (*Det-IR*), two Mrtf/Srf target genes (Jonchère et al., 2017), restored the growth of P-cad-expressing wings but not their circularity (Fig. S5).

Surprisingly, although P-cad appeared to enhance Mrtf–Srf signaling and reduced wing growth (Fig. 1E and Fig. 2A), Mrtf and Srf have been previously shown to enhance wing growth (Lyulcheva et al., 2008). Indeed, adult wings homozygous for the *Srf^2^* mutation were smaller than control wings expressing *GFP* under *nub*-Gal4 control. Conversely, overexpressing full-length *Mrtf* enhanced wing growth (Fig. S6A). Expression of a constitutively active form of *Mrtf* (*MrtfΔN*), with deletion of the N-terminus, which renders mammalian and *Drosophila* Mrtf nuclear and active (Miralles et al., 2003; Somogyi and Rørth, 2004), reduced wing size and circularity (Fig. S6A), reminiscent of the P-cad functional effect. These observations suggest that full-length Mrtf and MrtfΔN control distinct transcriptional targets. To confirm that P-cad potentiates Mrtf activity, we tested whether overexpressing full-length *Mrtf* or *MrtfΔN* further enhances the *nub>P-cad* adult wing phenotype. Accordingly, wings co-expressing *P-cad* and *MrtfΔN* were even smaller than those expressing each one alone (Fig. S6B). However, overexpressing full-length *Mrtf* did not further worsen the *nub>P-cad* phenotype. Instead, these wings were significantly bigger than those expressing P-cad alone (Fig. S6B). Thus, P-cad may affect Mrtf activity so that it acquires transcriptional properties reminiscent of the ones of *MrtfΔN*. Taken together, our observations are consistent with a role for P-cad in promoting constitutive Mrtf–Srf signaling activity.

### P-cad signals through the actin cytoskeleton

**Fig. 3.**
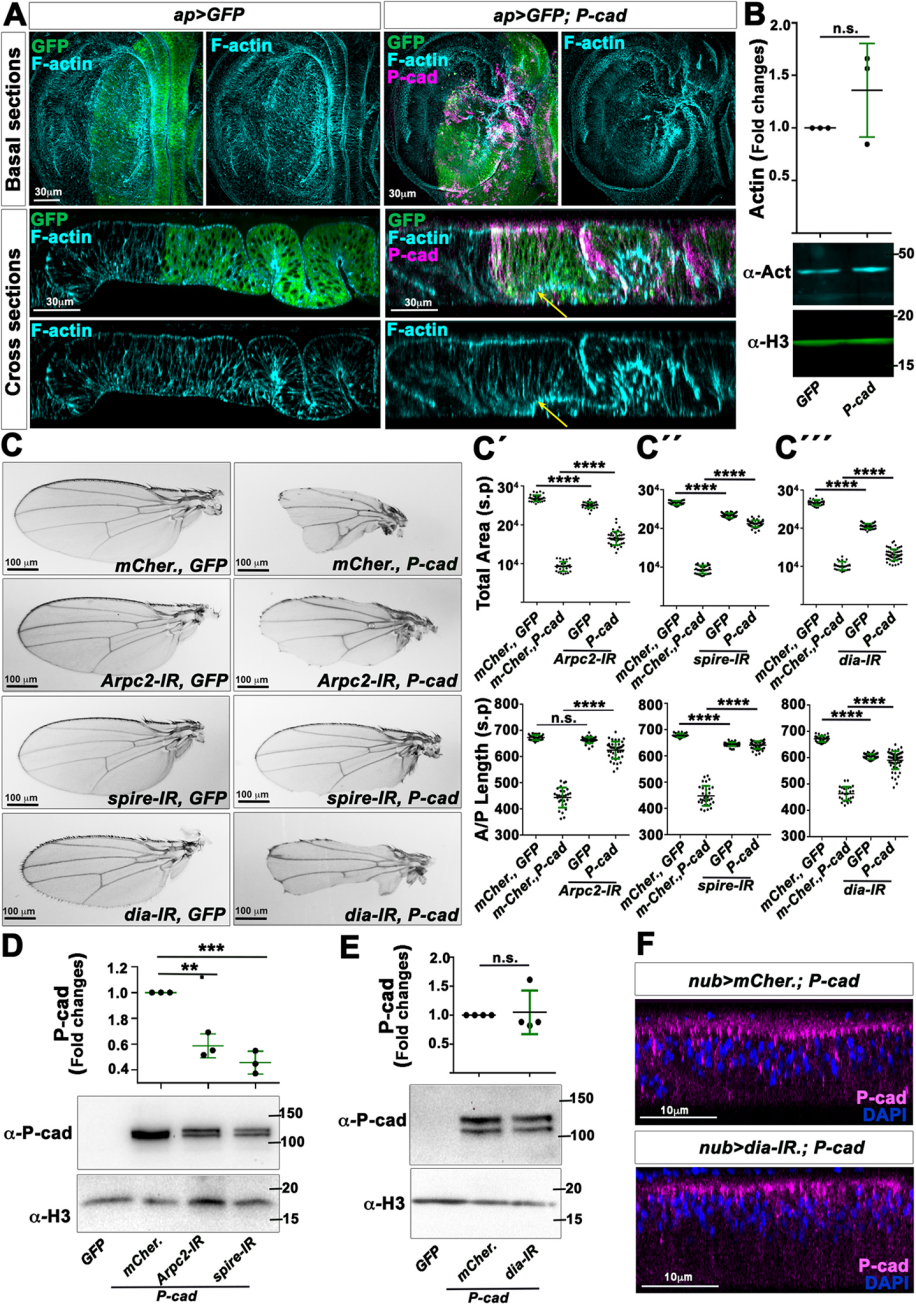
**Actin nucleation is required for P-cad functional effect in the fly wing.** (A) Standard confocal sections of the basal surfaces of third-instar wing imaginal discs (top) or cross-sections through wing imaginal discs (bottom) expressing UAS-*mCD8-GFP* (green) or UAS-*mCD8-GFP* (green) and UAS-*P-cad* under *ap-*Gal4 control and stained with Phalloidin (cyan) to mark the actin cytoskeleton and anti-P-cad (magenta). Scale bars: 30 µm. Staining for each genotype was performed three times. (B) (Bottom) Western blots of protein extracts from wing imaginal discs expressing the indicated constructs under *nub-*Gal4 control, blotted with anti-actin (α-Act), with anti-Histone 3 (α-H3) used as loading control. (Top) Quantification from three biological replicates of the ratio of actin levels normalized to α-H3 for the genotypes indicated. (C) Adult wings in which *nub-*Gal4 drives the indicated UAS-constructs. Scale bars: 100 µm. (C′-C‴) Quantifications of the total area (top) or anterior–posterior (A/P) length (bottom) of adult wings in which *nub-*Gal4 drives UAS-*CD8-GFP* and UAS-*mCherry* (*n*=29), or UAS-*mCherry* and UAS-*P-cad* (*n*=28), or UAS-*mCD8-GFP* and UAS-*Arpc2-IR* (*n*=34), or UAS-*P-cad* and UAS-*Arpc2-IR* (*n*=42) (C′); or UAS-*CD8-GFP* and UAS-*mCherry* (*n*=30), or UAS-*mCherry* and UAS-*P-cad* (*n*=31), or UAS-*mCD8-GFP* and UAS-*spire-IR* (*n*=44), or UAS-*P-cad* and UAS-*spire-IR* (*n*=47) (C″); or UAS-*CD8-GFP* and UAS-*mCherry* (*n*=31), or UAS-*mCherry* and UAS-*P-cad* (*n*=26), or UAS-*mCD8-GFP* and UAS-*dia-IR* (*n*=47), or UAS-*P-cad* and UAS-*dia-IR* (*n*=55) (C‴). Quantifications of control *nub>mCherry, GFP* and *nub>mCherry, P-cad* are from four or six independent crosses, respectively. Other quantifications are from one biological replicate. (D) (Bottom) Western blots of protein extracts from wing imaginal discs expressing the indicated UAS-constructs under *nub-*Gal4 control, blotted with anti-P-cad, with anti-α-H3 used as loading control. (Top) Quantification from three biological replicates of the ratio of P-cad levels normalized to α-H3 for the genotypes indicated. (E) (Bottom) Western blots of protein extracts from wing imaginal discs expressing the indicated UAS-constructs under *nub-*Gal4 control, blotted with anti-P-cad, with anti-α-H3 used as loading control. (Top) Quantification from four biological replicates of the ratio of P-cad levels normalized to α-H3 for the genotypes indicated. (F) Cross-sections of third-instar wing imaginal discs expressing the indicated UAS-constructs under *nub-*Gal4 control and stained with anti-P-cad (magenta) and DAPI (blue). Scale bars: 10 µm. Staining was performed once. Error bars indicate s.d.; n.s., non-significant; **P<0.01; ***P<0.001; ****P<0.0001. Statistical significance was calculated using one-way ANOVA with Tukey's multiple comparison (C) or unpaired two-tailed Student's *t*-tests (D,E).

As G-actin levels regulate the nucleus–cytoplasm shuttling of MRTF-A (Vartiainen et al., 2007; Pawłowski et al., 2010), we asked whether P-cad expression would affect the actin cytoskeleton. Accordingly, expressing *P-cad* and *GFP* with *ap*-Gal4 in the dorsal wing disc epithelium appeared to increase the pool of F-actin, which seemed to accumulate mainly on the basal surface of the disc epithelium (Fig. 3A). Yet, total actin levels were not significantly different between wing discs extracts expressing *GFP* only and those expressing *P-cad* and *GFP* under *ap-*Gal4 control (Fig. 3B). These observations suggest that P-cad reduces the pool of G-actin. We then tested whether reducing F-actin nucleation could suppress the P-cad-expressing wing phenotype. Indeed, knocking down components of the Arp2/3 complex or the tandem monomer binder *spire*, or even the formin *diaphanous* (*dia*), significantly restored the A/P length and size of *nub>P-cad*-expressing wings (Fig. 3C-C‴; Fig. S7). To test the possibility that the actin cytoskeleton rescues the P-cad wing phenotype by acting upstream of P-cad, we analyzed the effect of knocking down these actin nucleators on P-cad levels. Knocking down *Arpc2* or *spire* significantly reduced P-cad levels (Fig. 3D; Fig. S4B). In contrast, the levels of P-cad were not significantly different in wing disc extracts expressing *P-cad* and *dia-IR*, compared to those expressing *P-cad* and *mCherry* (Fig. 3E; Fig. S4C). Moreover, P-cad remained apically localized in wing disc epithelia expressing *dia-IR*, further suggesting that Dia is required downstream of P-cad to affect wing development. Although Arpc2 and Spire regulate P-cad protein stability, they could have additional functional effects downstream of P-cad. Consistent with this hypothesis, knocking down *Mrtf* or *Srf* did not further suppress the defects of wings expressing *P-cad* and *Arpc2-IR* or *spire-IR* (Fig. S8A). As the Mrtf–Srf pathway is well known to control the expression of many genes related to the actin cytoskeleton (Olson and Nordheim, 2010), we also tested whether Arpc2 or Spire promotes P-cad functional effects downstream of the Mrtf–Srf pathway. However, knocking down *Arpc2* or *spire* did not rescue the wing phenotype induced by expressing *MrtfΔN*. On the contrary, expressing *Arpc2-IR* or *spire-IR* further reduced the size or A/P length of these wings (Fig. S8B). Taken together, these observations suggest that the Arp2/3 complex and Spire stabilize P-cad, probably by regulating the actin cytoskeleton. In addition, actin regulation by the Arp2/3 complex, Spire and Dia might be involved downstream of P-cad, likely by enhancing the activity of the Mrtf–Srf signaling pathway.

### P-cad transiently accumulates in tamoxifen (TAM)-treated MCF10A-ER-Src cells

**Fig. 4.**
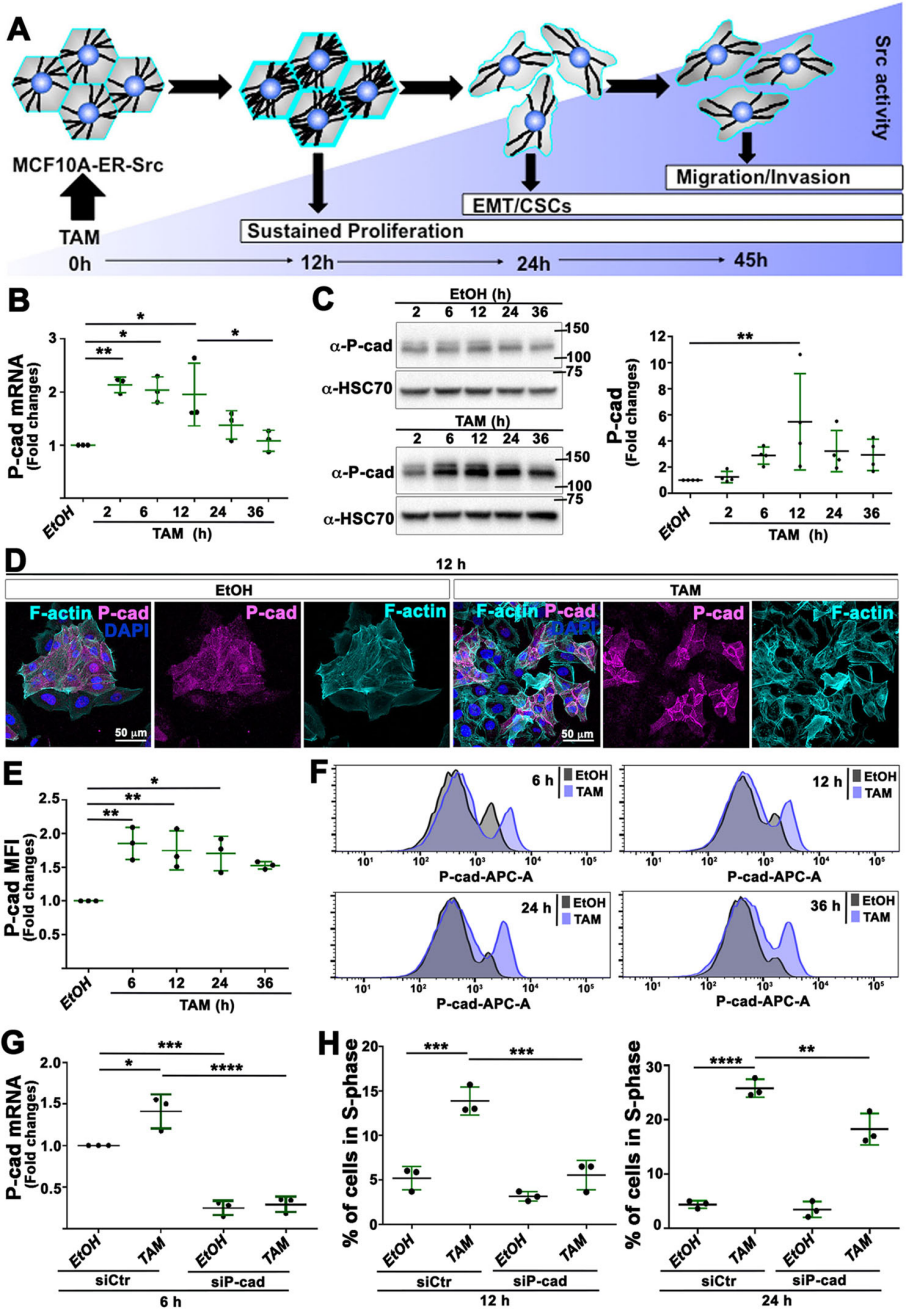
**P-cad transiently accumulates in tamoxifen (TAM)-treated MCF10A-ER-Src cells and is required to sustain their proliferation.** (A) Schematic of the series of events taking place during transformation of the MCF10A-ER-Src cell line treated with TAM. MCF10A-ER-Src cells transiently accumulate F-actin stress fibers 12 h after TAM treatment. In turn, stress fiber organization sustains cell proliferation, as well as further potentiates SRC activity. Cells then undergo epithelial–mesenchymal transition (EMT) and acquire cancer stem-like cell (CSC) properties 24 h after TAM treatment and gain invading abilities at 45 h of treatment (Iliopoulos et al., 2011; Tavares et al., 2017; Selvaggio et al., 2020). (B) Quantification from three biological replicates of *P-cad* mRNA levels in extracts from MCF10A-ER-Src cells treated with TAM for 2, 6, 12, 24 and 36 h, normalized to those in extracts from MCF10A-ER-Src cells treated with EtOH for the same time points. (C) (Left) Western blots of protein extracts from MCF10A-ER-Src cells treated with EtOH (top) or TAM (bottom) for 2, 6, 12, 24 or 36 h, blotted with anti-P-cad or anti-HSC70. (Right) Ratio from four biological replicates of P-cad levels between TAM- and EtOH-treated MCF10A-ER-Src cells for the same time points, normalized to HSC70. (D) Standard confocal sections of MCF10A-ER-Src cells treated with EtOH or TAM for 12 h, stained with Phalloidin (cyan), anti-P-cad (magenta) and DAPI (blue). Scale bars: 50 µm. (E) Quantification from three biological replicates of the median fluorescence intensity (MFI) of membrane-associated P-cad in MCF10A-ER-Src cells treated with TAM for 6, 12, 24 and 36 h, normalized to that in MCF10A-ER-Src cells treated with EtOH for the same time points. (F) Histograms of membrane-associated P-cad in MCF10A-ER-Src cells treated with TAM or EtOH for 6, 12, 24 and 36 h. (G) Quantification from three biological replicates of *P-cad* mRNA levels in extracts from MCF10A-ER-Src cells expressing *siCtr* or *siP-cad* and treated with EtOH or TAM for 6 h. (H) Quantification from three biological replicates of the percentage of MCF10A-ER-Src cells in S-phase, expressing *siCtr* or *siP-cad* and treated with EtOH or TAM for 12 h (left) or 24 h (right). Error bars indicate s.d.; *P<0.05; **P<0.01; ***P<0.001; ****P<0.0001. Statistical significance was calculated using unpaired two-tailed Student's *t*-test when comparing two conditions or one-way ANOVA with Tukey's multiple comparison in the case of multiple comparisons.

To validate the role of the Mrtf–Srf signaling pathway in promoting tumorigenic phenotypes downstream of P-cad in a human context, we used the mammary epithelial cell line MCF10A-ER-Src, which contains a fusion between the viral SRC kinase ortholog and the ligand-binding domain of the estrogen receptor (ER; also known as ESR), inducible with TAM treatment (Hirsch et al., 2009; Iliopoulos et al., 2009). cDNA microarray analysis indicates that TAM-treated MCF10A-ER-Src cells upregulate *P-cad* (*CDH3*) during the transition from normal to transformed cells, which involves the gain of sustained proliferative abilities 12 h after TAM treatment, EMT and the acquisition of stem-like properties 24 h after treatment, and invading abilities 36-45 h after TAM treatment (Hirsch et al., 2010; Iliopoulos et al., 2011; Tavares et al., 2017; Selvaggio et al., 2020). We have reported that the gain of self-sufficiency in growth properties and the progression towards a fully transformed state result from a transient increase in actomyosin-dependent cell stiffening during the first 12 h of TAM treatment, which is associated with a transitory accumulation of F-actin (Fig. 4A) (Tavares et al., 2017). We therefore checked whether this early accumulation of F-actin was associated with P-cad upregulation. The ratio of *P-cad* mRNA levels between cells treated with TAM and ethanol (EtOH) indicated that P-cad levels were significantly upregulated 2.13-fold 2 h after TAM treatment, maintained significant higher expression up to 12 h after treatment, and progressively dropped to initial levels 24 and 36 h after treatment (Fig. 4B). This transient increase in *P-cad* mRNA levels was translated into an increase in protein levels in cells treated with TAM, compared to in those treated with EtOH for the same time (Fig. 4C; Fig. S9A). Moreover, in cells treated with TAM for 12 h, P-cad strongly accumulated at the membrane in cells in contact with each other, which showed higher F-actin levels (Fig. 4D). Quantification of the median fluorescence intensity (MFI) of membrane-associated P-cad by fluorescence-activated cell sorting (FACS) showed that the levels of P-cad were significantly increased between 6 and 24 h after TAM treatment (Fig. 4E). Strikingly, a subpopulation of MCF10A-ER-Src cells was highly enriched with membrane-associated P-cad, starting 6 h after TAM treatment, compared to cells treated with EtOH (Fig. 4F). To determine whether the transient upregulation of P-cad was required for cellular transformation, we tested the consequence of knocking down P-cad using small interfering RNA (*siP-cad*) on the proliferation of TAM-treated MCF10A-ER-Src cells. MCF10A-ER-Src cells transfected with *siP-cad* and treated with EtOH or TAM for 6 h showed a 75% and 79% reduction, respectively, of *P-cad* mRNA levels, compared to EtOH or TAM-treated cells transfected with a siRNA control (*siCtr*) (Fig. 4G). In the absence of epidermal growth factor (EGF) and in low serum concentration, TAM-treated MCF10A-ER-Src cells showed a significant increase in the percentage of cells in S-phase of the cell cycle compared to that of EtOH-treated cells, 12 and 24 h after treatment. Knocking down P-cad significantly reduced the number of TAM-treated MCF10A-ER-Src cells in S-phase (Fig. 4H). Thus, P-cad accumulation correlates with the transient accumulation of F-actin in pre-malignant MCF10A-ER-Src cells and is required to sustain their proliferation.

### P-cad triggers MRTF-A–SRF signaling in TAM-treated MCF10A-ER-Src cells

**Fig. 5.**
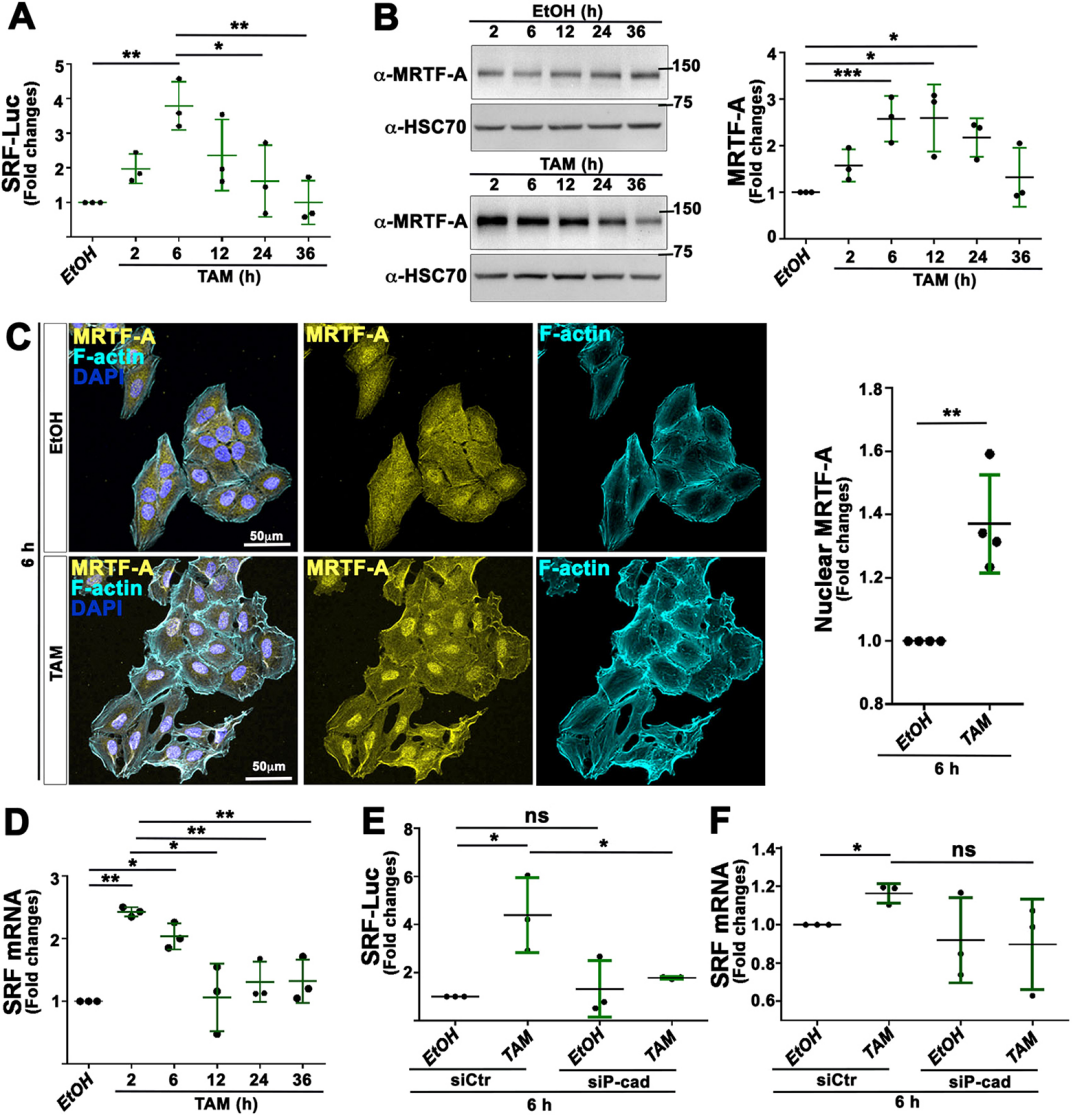
**Knocking down P-cad prevents the transient increase in SRF transcriptional activity in TAM-treated MCF10A-ER-Src cells.** (A) Quantification from three biological replicates of the fold changes in SRF Luciferase activity (SRF-Luc) between MCF10A-ER-Src cells treated with EtOH or TAM for 2, 6, 12, 24 and 36 h, transfected with the SRF-responsive-Luc reporter gene. (B) (Left) Western blots of protein extracts from MCF10A-ER-Src cells treated with EtOH or TAM for 2, 6, 12, 24 or 36 h, blotted with anti-MRTF-A or anti-HSC70. (Right) Quantification from three biological replicates of the ratio of MRTF-A levels between TAM- and EtOH-treated MCF10A-ER-Src cells for the same time points, normalized to HSC70. (C) (Left) Standard confocal sections of MCF10A-ER-Src cells treated with EtOH or TAM for 6 h, stained with Phalloidin (cyan blue), anti-MRTF-A (yellow) and DAPI (blue). Scale bars: 50 µm. (Right) Quantification from four biological replicates of the intensity of nuclear MRTF-A in MCF10A-ER-Src cells treated with EtOH or TAM for 6 h. (D) Quantification from three biological replicates of *SRF* mRNA levels in extracts from MCF10A-ER-Src cells treated with TAM for 2, 6, 12, 24 and 36 h, normalized to those in extracts from MCF10A-ER-Src cells treated with EtOH for the same time points. (E) Quantifications from three biological replicates of the fold changes in SRF-Luc activity between MCF10A-ER-Src cells transfected with the SRF-responsive-Luc reporter gene, expressing *siCtr* or *siP-cad* and treated with EtOH and TAM for 6 h. (F) Quantification from three biological replicates of *SRF* mRNA levels in extracts from MCF10A-ER-Src cells expressing *siCtr* or *siP-cad* and treated with EtOH or TAM for 6 h. Error bars indicate s.d.; ns, non-significant; *P<0.05; **P<0.01; ***P<0.001. Statistical significance was calculated using unpaired two-tailed Student's *t*-test when comparing two conditions or one-way ANOVA with Tukey's multiple comparison in the case of multiple comparisons.

To determine whether the transient accumulation of F-actin and P-cad was also associated with a boost of SRF transcriptional activity, we measured the activity of a SRF Luciferase (SRF-Luc) reporter, which contains a Luciferase construct controlled by three SRF binding sites (Posern et al., 2002). Temporal analysis showed that Luciferase activity rose up to 3.8-fold during the first 6 h of TAM treatment, before dropping 36 h after treatment to levels similar to those of EtOH-treated cells (Fig. 5A). This increase in SRF transcriptional activity was also associated with the transient accumulation of MRTF-A, as revealed by western blotting, during the first 24 h of TAM treatment (Fig. 5B), as well as with the nuclear accumulation of MRTF-A in MCF10A-ER-Src cells treated with TAM for 6 h (Fig. 5C). Furthermore, the expression of *SRF*, which has been previously identified as a direct MRTF-A/SRF target gene (Selvaraj and Prywes, 2004), was also transiently upregulated 2-6 h after TAM treatment (Fig. 5D).

We then knocked down P-cad in TAM-treated cells to test whether the transient increase in SRF transcriptional activity required P-cad. Knocking down P-cad in TAM-treated cells significantly abrogated the transient increase in SRF-dependent Luciferase activity (Fig. 5E). *SRF* mRNA levels were also reduced upon P-cad silencing, although this decrease was not statistically significant (Fig. 5F). Taking these results together, we conclude that the transient accumulation of P-cad in TAM-treated MCF10A-ER-Src cells triggers a temporal increase in MRTF-A–SRF signaling prior to the acquisition of malignant features.

### Actin polymerization induces SRF activity in TAM-treated MCF10A-ER-Src cells

**Fig. 6.**
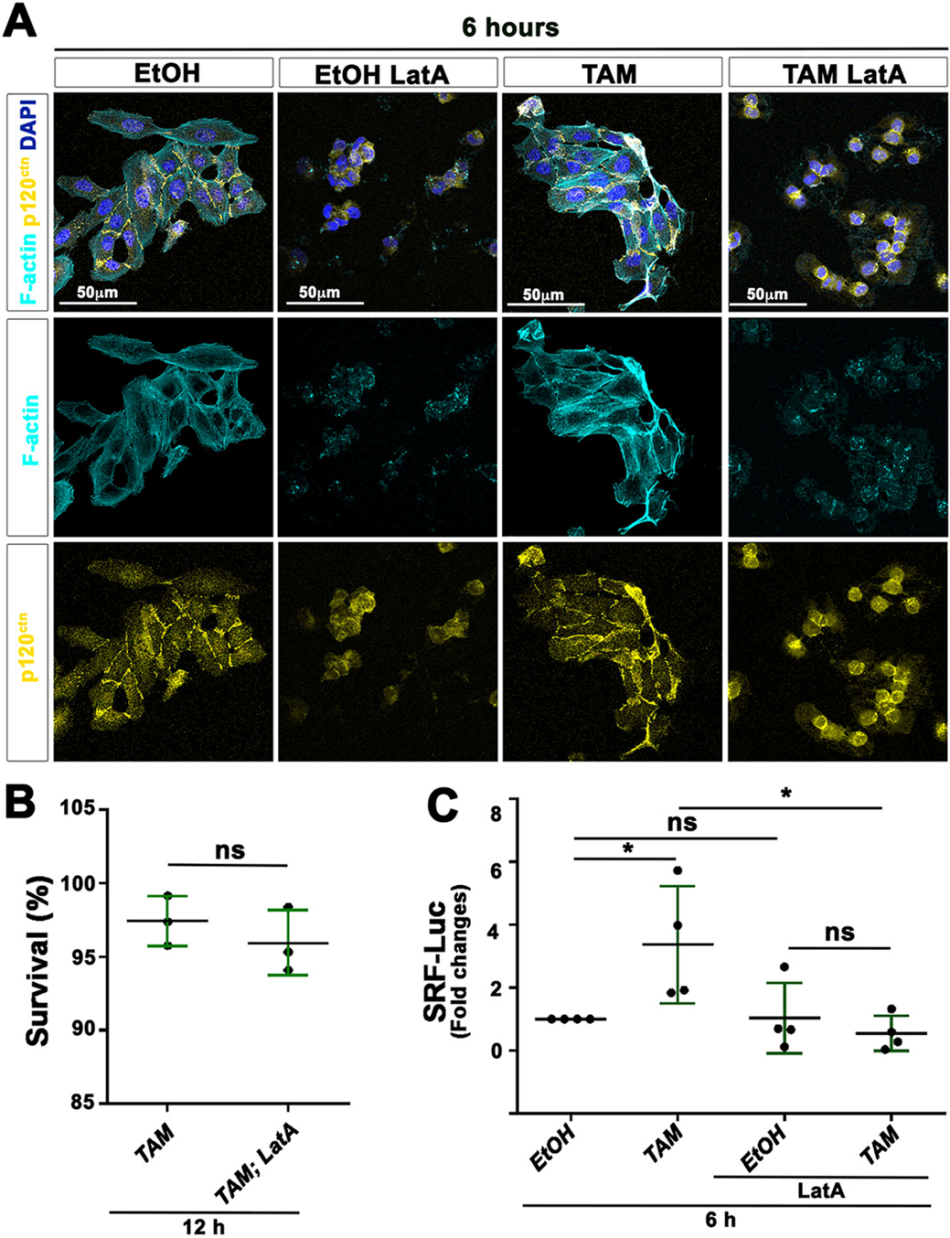
**Latrunculin A (LatA) inhibits SRF transcriptional activity in MCF10A-ER-Src cells treated with TAM for 6 h.** (A) Standard confocal sections of MCF10A-ER-Src cells treated with EtOH or TAM for 6 h, in the presence or absence of LatA, stained with Phalloidin (cyan) to mark F-actin, anti-p120^ctn^ (yellow) to stain cell–cell adhesions and DAPI (blue). Scale bars: 50 µm. (B) Quantification from three biological replicates of the percentage of viable MCF10A-ER-Src cells treated with TAM for 12 h, in the presence or absence of LatA. (C) Quantifications from four biological replicates of the fold changes in Luciferase activity between MCF10A-ER-Src cells treated with EtOH or TAM for 6 h in the presence or absence of LatA, transfected with the SRF-responsive-Luc reporter gene. Error bars indicate s.d.; ns, non-significant; *P<0.05. Statistical significance was calculated using unpaired two-tailed Student's *t*-test when comparing two conditions or one-way ANOVA with Tukey's multiple comparison in the case of multiple comparisons.

TAM treatment promotes a transient increase in actin polymerization in MCF10A-ER-Src cells within the first 12 h (Tavares et al., 2017). Thus, we probed whether the increased MRTF-A–SRF signaling activity depended on transient F-actin accumulation, by treating cells with Latrunculin A (LatA), which blocks the dissociation of the G-actin:MRTF-A complex (Sotiropoulos et al., 1999; Miralles et al., 2003). MCF10A-ER-Src cells, which maintained p120 catenin (p120^ctn^) at cell–cell contacts, showed higher F-actin levels when treated with TAM for 6 h. In contrast, LatA markedly reduced F-actin levels in both EtOH- and TAM-treated cells (Fig. 6A), without affecting cell viability, which was maintained high, at ∼96-97%, 12 h after treatment (Fig. 6B). Although LatA treatment had no significant effect on SRF-Luc activity in MCF10A-ER-Src cells treated with EtOH for 6 h, it abrogated the Luciferase activity in cells treated with TAM for 6 h (Fig. 6C). Thus, actin polymerization is required to induce the transient increase in SRF signaling activity.

### MRTF-A triggers malignant transformation in TAM-treated MCF10A-ER-Src cells

**Fig. 7.**
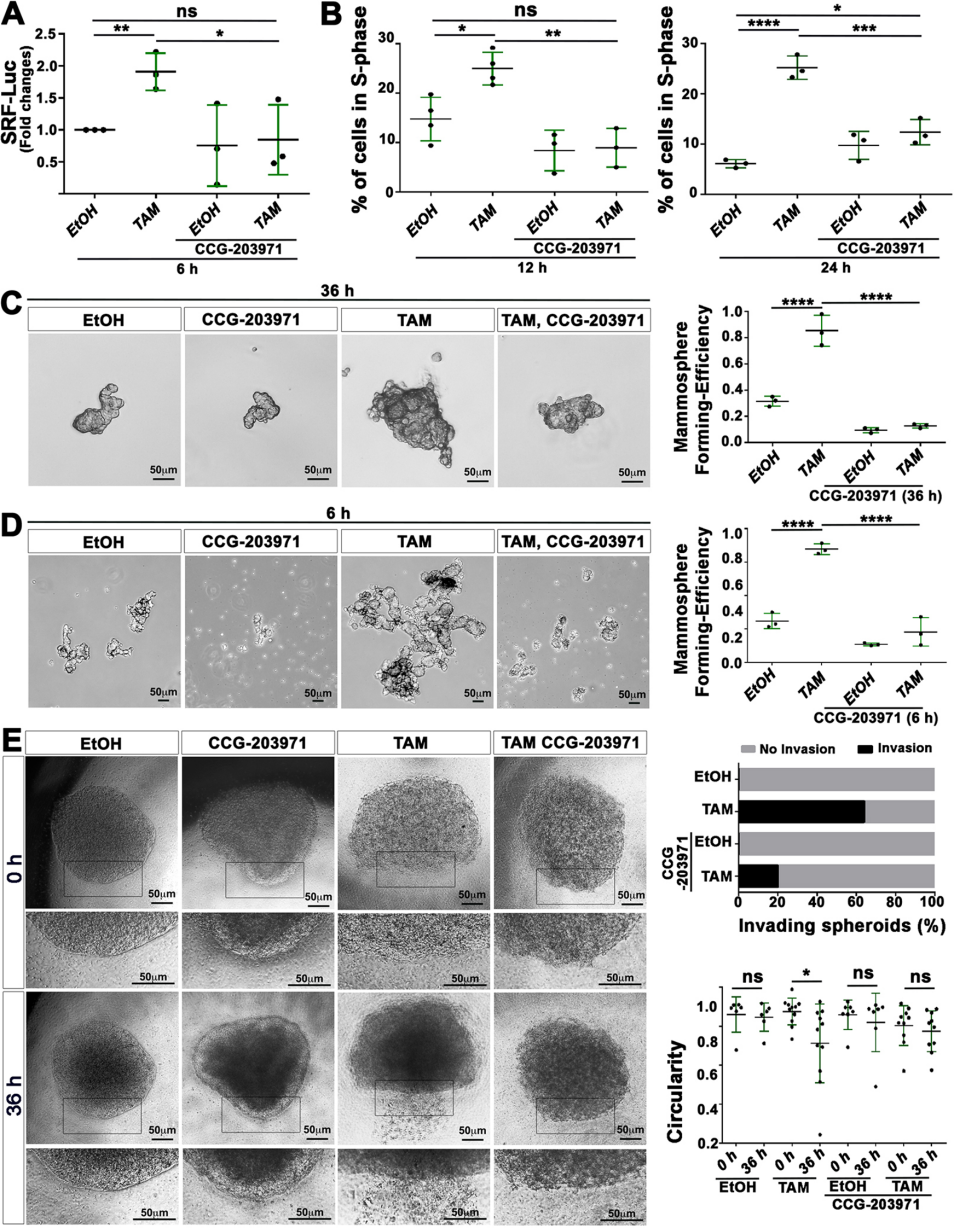
**CCG-203971 reduces the ability of TAM-treated MCF10A-ER-Src cells to sustain proliferation, to grow mammospheres and to invade in collagen.** (A) Quantification from three biological replicates of the fold changes in Luciferase activity between MCF10A-ER-Src cells treated with EtOH or TAM, in the presence or absence of CCG-203971, transfected with the SRF-responsive-Luc reporter gene. (B) Quantification from four (left) or three (right) biological replicates of the percentage of MCF10A-ER-Src cells in S-phase, treated for 12 h (left) or 24 h (right) with EtOH or TAM in the presence or absence of CCG-203971. (C,D) (Left) Representative images of MCF10A-ER-Src mammospheres treated for 36 h (C) or 6 h (D) with EtOH or TAM, in the presence or absence of CCG-203971. Scale bars: 50 μm. (Right) Quantification from three biological replicates of the mammosphere-forming efficiency of MCF10A-ER-Src mammospheres treated with EtOH or TAM for 36 h (C) or 6 h (D), in the presence or absence of CCG-203971. (E) (Left) Representative images of MCF10A-ER-Src spheroids in collagen before treatment (0 h) or after 36 h of treatment with EtOH or TAM, in the presence or absence of CCG-203971. The lower images are magnifications of the region delimited by the black outline in the top images. Scale bars: 50 μm. (Top right) Quantification from two biological replicates of the percentage of invading MCF10A-ER-Src spheroids treated with EtOH in the absence (*n*=6) or presence (*n*=7) of CCG-203971 or treated with TAM in the absence (*n*=11) or presence (*n*=10) of CCG-203971. (Bottom right) Quantification from two biological replicates of the circularity of MCF10A-ER-Src spheroids in collagen before treatment (0 h) and after 36 h of treatment with EtOH in the absence (*n*=6) or presence (*n*=7) of CCG-203971, or before treatment (0 h) and after 36 h of treatment with TAM in the absence (*n*=11) or presence (*n*=10) of CCG-203971. Error bars indicate s.d.; ns, non-significant; *P<0.05; **P<0.01; ***P<0.001; ****P<0.0001. Statistical significance was calculated using unpaired two-tailed Student's *t*-test (E) or one-way ANOVA with Tukey's multiple comparison (A-D).

To explore the role of the transient increase in MRTF-A–SRF signaling activity prior to the acquisition of malignant features, we used the small-molecule inhibitor CCG-203971, which has been reported to block the nuclear localization and activity of MRTF-A without causing cellular toxicity (Bell et al., 2013; Haak et al., 2017). We first confirmed that the expression of the minimal SRF-dependent Luciferase reporter gene was dependent on MRTF-A activity. Accordingly, MCF10A-ER-Src cells co-treated with CCG-203971 and TAM failed to increase SRF-dependent Luciferase activity, compared to those that were treated with TAM only (Fig. 7A). We then tested the consequences of blocking MRTF-A activity on the ability of TAM-treated MCF10A-ER-Src cells to acquire transformed features. Like P-cad (Fig. 4H), MRTF-A activity is required for TAM-treated cells to gain self-sufficiency in growth properties, as CCG-203971 abrogated their proliferative advantage 12 and 24 h after TAM treatment (Fig. 7B). As previously reported (Iliopoulos et al., 2011), MCF10A-ER-Src cells treated with TAM for 36 h formed significantly more mammospheres than those treated with EtOH. Blocking MRTF-A activity during the 36 h of treatment using CCG-203971 markedly reduced the mammosphere-forming ability of TAM-treated cells (Fig. 7C), indicating that MRTF-A activity is required for TAM-treated cells to acquire self-renewing abilities. The mammosphere-forming ability of TAM-treated cells depends on the transient increase in MRTF-A activity in pre-malignant cells, as inhibiting MRTF-A activity during the first 6 h of TAM treatment was sufficient to abrogate mammosphere formation (Fig. 7D).

We then assessed the effect of CCG-203971 on the ability of TAM-treated MCF10A-ER-Src cells to invade in collagen. Although MCF10A-ER-Src spheroids treated with EtOH for 36 h maintained a round shape with well-defined borders, those that were treated with TAM for the same time displayed irregular borders with cells invading into the collagen matrix (Fig. 7E). CCG-203971 reduced the number of invading TAM-treated spheroids and restored their circularity. Altogether, these observations suggest that the P-cad-dependent increase in MRTF-A activity provides proliferative abilities to TAM-treated MCF10A-ER-Src cells and is required for malignant transformation.

## DISCUSSION

Here, we provide evidence that the humanized P-cad fly model we have generated is a clinically relevant platform for the identification of P-cad effectors. In agreement with observations reported in human cells (Kudo et al., 2014), we show that P-cad could establish *trans*-junctional homophilic interactions when expressed in *Drosophila* epithelia. Moreover, P-cad induces cell invasion and appears to signal through DE-cad, Src and Integrin in the wing disc epithelium, similar to its functional effect in breast cancer cells (Ribeiro et al., 2013, 2018; Vieira et al., 2014). Furthermore, P-cad expression induces major defects in undifferentiated/progenitor cells of the eye and wing discs, whereas in epithelial cells undergoing differentiation, P-cad effects are much weaker. Consistent with these observations, P-cad promotes the maintenance of an undifferentiated state in the normal mammary gland, but has no apparent effect when expressed in differentiated mammary epithelial cells (Radice et al., 1997, 2003). Yet, our P-cad fly model does not mimic all outcomes caused by P-cad in breast cancer cells. In these cells, P-cad promotes tumorigenic capacity (Ribeiro et al., 2013). In contrast, when expressed in the proliferating wing blade or eye disc primordium, P-cad prevents tissue growth. These distinct P-cad-dependent functional outcomes can result from cellular- and tissue-specific responses. Accordingly, the wing blade domain has been described as a ‘tumor coldspot’ unfavorable for tumor initiation due to the tissue-intrinsic local cytoarchitecture (Tamori et al., 2016).

Our humanized fly model revealed Mrtf and Srf as modifiers of the wing phenotype produced by P-cad overexpression. Like expressing P-cad, expressing a constitutively active form of *Mrtf* reduces wing growth. As P-cad levels or localization are not affected by knocking down Mrtf and Srf, and because P-cad induces Mrtf nuclear translocation, we propose that the MRTF-A–SRF axis is a key P-cad effector pathway in human cells. We have validated this hypothesis using a mammary epithelial cell line, which recapitulates molecular events taking place during the transition from a normal to a malignant state. Although P-cad overexpression has been implicated in sustaining malignant phenotypes in fully transformed breast cancer cells (Vieira and Paredes, 2015), our observations suggest that P-cad could also play a major role in the very early stages of breast carcinogenesis by promoting a transient boost of MRTF-A–SRF signaling pathway activity, essential for the acquisition of pre-malignant and malignant phenotypes. We show that one of the first responses to SRC activation in MCF10A-ER-Src cells is the transient accumulation of P-cad, which correlates with MRTF-A accumulation, its nuclear translocation, and the upregulation of SRF itself and of a minimal SRF transcriptional reporter gene. Moreover, knocking down P-cad or preventing MRTF-A nuclear translocation with CCG-203971 (Bell et al., 2013; Haak et al., 2017) impedes SRF transcriptional activity. Furthermore, we show that the transient boost of P-cad and MRTF-A activity provides proliferative advantages to TAM-treated MCF10A-ER-Src cells. In addition, MRTF-A nuclear translocation is required for the malignant transformation of these cells. Consistent with our observations, overexpressing MRTF-A in untransformed MCF10A acini is sufficient to induce pre-malignant spheroid formation caused by the dysregulation of cell cycle genes and EMT (Seifert and Posern, 2017). This mechanism may also be in place in other cancer subtypes, including those of the pancreas. In these cancer cells, P-cad has tumor-promoting effects (Sakamoto et al., 2015). Moreover, the overexpression of MRTF-A in normal pancreatic cells induces stem cells features and EMT (Song et al., 2016).

Interestingly, overexpressing full-length *Mrtf* or *MrtfΔN*, which constitutively localizes to the nucleus and promotes SRF transcriptional activity in fly and mammals (Miralles et al., 2003; Somogyi and Rørth, 2004; Vartiainen et al., 2007), leads to opposite wing phenotypes, suggesting that they control distinct targets. Our observations suggest that P-cad affects Mrtf so that it may acquire transcriptional properties reminiscent of the ones of a constitutive active form of Mrtf. Although further studies are required to validate these hypotheses, the status or amount of Mrtf in the nucleus could determine its ability to control the expression of various genes through distinct mechanisms involving Srf or independently of Srf, by binding directly to DNA or to other transcription factors, as previously reported in mammalian cells (Asparuhova et al., 2011; Gurbuz et al., 2014; Gau and Roy, 2018). P-cad potentiates the expression of a minimal SRF reporter in pre-malignant MCF10A-ER-Src cells, suggesting that the P-cad-dependent nuclear accumulation of MRTF-A promotes cellular transformation in part through SRF. In addition, the P-cad-dependent increase in MRTF-A activity could also affect gene expression independently of SRF. Consistent with this possibility, microarray analysis indicates that tenascin C, the expression of which is regulated directly by MRTF-A, is upregulated in TAM-treated MCF10A-ER-Src cells (Hirsch et al., 2010; Asparuhova et al., 2011). Moreover, MRTF-A triggers the expression of a specific set of pro-proliferative genes, independently of SRF (Gurbuz et al., 2014). MRTF-A activity could, in turn, regulate expression of P-cad, the promoter of which contains a single-nucleotide polymorphism in the SRF CArG binding site (Benson et al., 2011). Although activation of the P-cad–actin–MRTF-A–SRF pathway is a transient event during the early stages of cellular transformation, a small population of TAM-treated MCF10A-ER-Src cells could maintain high P-cad-dependent MRTF-A/SRF activity, as some cells retain high levels of membrane-associated P-cad 24 and 36 h after TAM treatment. Consistent with a role for MRTF-A–SRF signaling in mediating P-cad functional effects in fully transformed cells, MRTF-A/SRF has been shown to play an important role in the metastatic lung colonization of breast cancer cells and in drug resistance of basal cell carcinoma (Medjkane et al., 2009; Whitson et al., 2018).

We have previously reported that MCF10A-ER-Src cells undergo a transient increase in F-actin assembly, as well as show actomyosin-dependent cell stiffening during the first 12 h of TAM treatment. Preventing cell stiffening in these cells, without interfering with F-actin accumulation, prevents ERK (also known as MAPK)-dependent cell proliferation, reduces SRC activation and restricts the progression towards a fully transformed state (Tavares et al., 2017). We now provide evidence that the transient increase in F-actin assembly, triggered in part by P-cad, induces MRTF-A/SRF-dependent malignant transformation. First, P-cad appears to cause F-actin accumulation in the wing disc epithelium but does not affect the total pool of actin. Accordingly, P-cad affects the actin cytoskeleton in breast cancer cells (Ribeiro et al., 2016, 2018). Second, P-cad requires the actin-nucleating activity of the Arp2/3 complex, Spire and Dia to affect wing development. Third, blocking the dissociation of the G-actin:MRTF-A complex using LatA (Sotiropoulos et al., 1999; Miralles et al., 2003) impedes SRF transcriptional activity in TAM-treated cells. The increase in actomyosin-dependent cell stiffening could also enhance MRTF-A activity, as MRTF-A has been shown to respond to mechanical stress (Chan et al., 2010; Kuwahara et al., 2010; Seifert and Posern, 2017). P-cad could promote actin nucleation through activation of Rho-GTPases, like RAC1 and CDC42, which are well-known P-cad effectors and regulators of MRTF-A nuclear translocation and SRF transcription (Miralles et al., 2003; Plutoni et al., 2016; Gau and Roy, 2018). Thus, during the early stage of breast cancer, major alterations to the actin cytoskeleton could control signaling pathways through distinct mechanisms: an increase in F-actin assembly would induce signaling pathways, such as MRTF-A–SRF, while actomyosin activity would trigger the mechanical induction of signaling components, such as ERK and SRC, highlighting the key contribution of actin cytoskeleton regulation to carcinogenesis.

## MATERIALS AND METHODS

### Generation of P-cad transgenic fly

The Gateway Cloning System (Life Technologies) was used to create pUASp-P-cad. The CDH3/P-cad coding sequence was first cloned into pENTR, and pUAS-P-cad was then generated by LR clonase II-mediated recombination of pENTR-PCAD into a modified Gateway vector pPW-attB (Figueiredo et al., 2021). The pUASp-PCAD transgene was then inserted into the *attP40* landing site by site-specific transgenesis (BestGene Inc.). The original pUASp-PCAD transgenic line is available upon request.

### Fly strains and genetics

Fly stocks used were *nub-*Gal4 (Calleja et al., 1996); *ap-*Gal4, *eye-*Gal4, *GMR*-Gal4, *Sgs3-*Gal4 [Bloomington Drosophila Stock Center (BDSC) #6870]; *E-cad::GFP* (Huang et al., 2009); UAS-*p35* (Hay et al., 1994); UAS-*Diap1* (Ryoo et al., 2004); UAS-*DE-cad-IR^HMS00693^*, UAS-*Src64B-IR^JF03234^* (*Src64B-IR* #1), UAS-*Src64B-IR^HMC0332^* (*Src64B-IR* #2), UAS-*αPS2-IR^JF02695^*, UAS-*Mrtf-IR^JF02220^*, UAS-*Srf-IR^JF02319^*, *tub*-*Mrtf.3XGFP* (BDSC #58445); UAS-*MrtfΔN*, UAS-*MrtfS3*; *bs^2^* (*Srf^2^*), UAS-*MESK2-IR^JF03312^*, UAS-*Det-IR^GL00572^*, UAS-*spire-IR^JF03233^*, UAS-*Arpc2-IR^JF02845^*, UAS-*dia-IR^HM05027^*, UAS-*Arp2-IR^JF02785^*, UAS-*Arpc3A-IR^JF02370^*, UAS-*Arpc3B-IR^JF02679^*, UAS-*Arpc4-IR^JF01683^*, UAS-*Arpc5-IR^JF03147^* (BDSC). All crosses were maintained at 25°C. Adult wings are from 1- to 2-day-old females.

### Fly crosses

Fly crosses used in figure panels are as follows: *hs*FLP; *tub*-FRT stop FRT Gal4; UAS::*GFP/CyO X* UAS-*P-cad* and *nub-*Gal4, UAS-*P-cad/Cyo* X *DE-cad::GFP* (Fig. 1B); *ap-*Gal4, *DE-cad::GFP/CyO X* UAS*-mCherry* and *ap-*Gal4, *DE-cad::GFP/CyO X* UAS*-P-cad* (Fig. 1C); *nub-*Gal4; UAS-*mCD8-GFP* X w^1118^, *nub-*Gal4; UAS-*mCD8-GFP* X UAS-*DE-cad-IR^HMS00693^* and *nub-*Gal4; UAS-*P-cad* X UAS-*DE-cad-IR^HMS00693^* (Fig. 1D); *nub-*Gal4; UAS-*mCD8-GFP* X w^1118^, *nub-*Gal4, UAS-*P-cad/CyO* X UAS-*mCherry* and *nub-*Gal4, UAS-*P-cad/CyO* X UAS-*DE-cad-IR^HMS00693^* (Fig. 1E-G); *ap-*Gal4/*CyO*; UAS-*mCD8-GFP* X w^1118^ (Fig. 1H); *ap-*Gal4/*CyO*; UAS-*mCD8-GFP* X UAS-*P-cad* (Fig. 1I); *nub-*Gal4; UAS-*mCD8-GFP* X UAS*-mCherry*, *nub-*Gal4, UAS-*P-cad/CyO* X UAS-*mCherry*, *nub-*Gal4; UAS-*mCD8-GFP* X UAS-*Src64B-IR^JF03234^* (*Src64B-IR* #1), *nub-*Gal4, UAS-*P-cad/CyO* X UAS-*Src64B-IR^JF03234^* (*Src64B-IR* #1), *nub-*Gal4; UAS-*mCD8-GFP* X UAS-*Src64B-IR^HMC03327^* (*Src64B-IR* #2), *nub-*Gal4, UAS-*P-cad/CyO* X UAS-*Src64B-IR^HMC03327^* (*Src64B-IR* #2), *nub-*Gal4; UAS-*mCD8-GFP* X UAS-*αPS2-IR^JF02695^* and *nub-*Gal4, UAS-*P-cad/CyO* X UAS-*αPS2-IR^JF02695^* (Fig. 1J-L); *nub-*Gal4; UAS-*mCD8-GFP* X UAS*-mCherry*, *nub-*Gal4, UAS-*P-cad/CyO* X UAS-*mCherry*, *nub-*Gal4; UAS-*mCD8-GFP* X UAS-*Mrtf-IR^JF02220^*, *nub-*Gal4, UAS-*P-cad/CyO* X UAS-*Mrtf-IR^JF02220^*, *nub-*Gal4; UAS-*mCD8-GFP* X UAS-*Srf-IR^JF02319^* and *nub-*Gal4, UAS-*P-cad/CyO* X UAS-*Srf-IR^JF02319^* (Fig. 2A); *nub-*Gal4; UAS-*mCD8-GFP* X w^1118^ (lane 1), *nub-*Gal4, UAS-*P-cad/CyOTb* X UAS-*mCherry* (lane 2), *nub-*Gal4, UAS-*P-cad/CyOTb* X UAS-*Mrtf-IR^JF02220^* (lane 3) and *nub-*Gal4, UAS-*P-cad/CyOTb* X UAS-*Srf-IR^JF02319^* (lane 4) (Fig. 2B); *nub-*Gal4, UAS-*P-cad/CyO* X UAS-*mCherry*, *nub-*Gal4, UAS-*P-cad/CyO* X UAS-*Mrtf-IR^JF02220^* and *nub-*Gal4, UAS-*P-cad/CyO* X UAS-*Srf-IR^JF02319^* (Fig. 2C); *tub*-Mrtf.3XGFP; *Sgs3*-Gal4 X UAS-*P-cad* and *tub*-Mrtf.3XGFP X *Sgs3*-Gal4 (Fig. 2D); *ap-*Gal4/*CyO*; UAS-*mCD8-GFP* X w^1118^ and *ap-*Gal4/*CyO*; UAS-*mCD8-GFP* X UAS-*P-cad* (Fig. 3A,B); *nub-*Gal4; UAS-*mCD8-GFP* X UAS*-mCherry*, *nub-*Gal4, UAS-*P-cad/CyO* X UAS-*mCherry*, *nub-*Gal4; UAS-*mCD8-GFP* X UAS-*Arpc2-IR^JF02845^*, *nub-*Gal4, UAS-*P-cad/CyO* X UAS-*Arpc2-IR^JF02845^*, *nub-*Gal4; UAS-*mCD8-GFP* X UAS-*spire-IR^JF03233^*, *nub-*Gal4, UAS-*P-cad/CyO* X UAS-*spire-IR^JF03233^*, *nub-*Gal4; UAS-*mCD8-GFP* X UAS-*dia-IR^HM05027^* and *nub-*Gal4, UAS-*P-cad/CyO* X UAS-*dia-IR^HM05027^* (Fig. 3C-C‴); *nub-*Gal4; UAS-*mCD8-GFP* X w^1118^ (lane 1), *nub-*Gal4, UAS-*P-cad/CyOTb* X UAS-*mCherry* (lane 2), *nub-*Gal4, UAS-*P-cad/CyOTb* X UAS-*Arpc2-IR^JF02845^* (lane 3) and *nub-*Gal4, UAS-*P-cad/CyOTb* X UAS-*spire-IR^JF03233^* (lane 4) (Fig. 3D); *nub-*Gal4; UAS-*mCD8-GFP* X w^1118^ (lane 1), *nub-*Gal4, UAS-*P-cad/CyOTb* X UAS-*mCherry* (lane 2) and *nub-*Gal4, UAS-*P-cad/CyOTb* X UAS-*dia-IR^HM05027^* (lane 3) (Fig. 3E); *nub-*Gal4, UAS-*P-cad/CyO* X UAS-*mCherry* and *nub-*Gal4, UAS-*P-cad/CyO* X UAS-*dia-IR^HM05027^* (Fig. 3F).

The FLPout system (Bischof and Basler, 2008) was used to induce clonal P-cad overexpression (Fig. 1A). UAS-*P-cad* transgenic lines were crossed with y,w, *hs*Flp; *tub*-FRT-stop-FRT-Gal4 UAS:*GFP*/CyO, and the progeny (y,w, *hs*Flp; UAS-*P-cad/tub*-FRT-stop-FRT-Gal4, UAS:*GFP*) was heat shocked to randomly induce Flippase-mediated removal of the Flippase recognition target (FRT) cassette.

### Cell culture conditions and drug treatments

The MCF10A-ER-Src cell line was kindly provided by Kevin Struhl (Hirsch et al., 2009). Cells were grown in a humidified incubator at 37°C, under a 5% CO_2_ atmosphere in complete growth medium (CGM), composed of DMEM/F12 growth medium (Gibco, 11039-047), supplemented with 5% charcoal-stripped horse serum (CSHS; Gibco, 16050-122), 20 ng/ml human EGF (Peprotech, AF-100-15), 0.5 µg/ml hydrocortisone (Sigma-Aldrich, H0888), 100 ng/ml cholera toxin from *Vibrio cholerae* (Sigma-Aldrich, C8052), 10 µg/ml insulin (Sigma-Aldrich, I9278) and 0.5 µg/ml puromycin (Merck, 540411). The presence of mycoplasma contamination was tested by PCR using the primers MGS0 5′-TGCACCATGTGTCACTCTGTTAACCTC-3′ and GPO1 5′-ACTCCTACGGGAGGCAGCAGTA-3′, which amplify the 16S ribosomal RNA genes of the Mollicutes class of mycoplasma. The latest test for mycoplasma contamination was performed on 25 January 2019 on cells frozen at passage 12. These cells were used for all experiments. To treat cells with 4OH-TAM or EtOH, 50% confluent cells were plated and allowed to adhere for at least 24 h before treatment with 1 µM 4OH-TAM (Merck, H7904) or with an identical volume of EtOH for the indicated time. To assess the effect of preventing actin polymerization or MRTF-A nuclear translocation, cells were grown in CGM for 12-24 h, washed and co-treated with 4OH-TAM or EtOH in the presence or absence of 0.5 µM LatA (Labclinics, 10010630) or of 40 µM CCG-203971 (Merck, SML1422) in restricted growth medium (RGM), composed of DMEM/F12, 0.5% CSHS, 0.5 µg/ml hydrocortisone, 100 ng/ml cholera toxin and 10 µg/ml insulin for the time points indicated.

### Real-time PCR analysis

RNAs were extracted using a NZY Total RNA Isolation Kit (NZYTech, MB13402). For first-strand cDNA synthesis (NZYTech, MB125), 0.5 µg purified RNA samples were used, according to the manufacturer's instructions. Real-time PCR was performed on 5 ng/µl cDNA using iTaq Universal SYBR Green Supermix (Bio-Rad, 64361172) according to the manufacturer's instructions. Quantitative RT-PCR was performed in triplicates using a CFX Touch Real Time detector system, and relative fold change was calculated using ddCT method. Primer sequences used are listed in Table S1.

### Immunofluorescence analysis

For immunofluorescence of the follicular epithelium, *Drosophila* ovaries were dissected in Schneider's insect medium (Sigma-Aldrich) with 10% fetal bovine serum (FBS) and fixed in 4% paraformaldehyde. Following washing steps with 0.05% Tween-20 in PBS (PBS-T), and blocking with 10% bovine serum albumin (BSA) in PBS-T, ovaries were incubated overnight with rabbit anti-P-cad (1:40; Cell Signaling Technology, 2130S) and rat anti-E-cad [1:20; Developmental Studies Hybridoma Bank (DSHB)]. Secondary antibodies used were Alexa Fluor 568 goat anti-mouse (1:300; Life Technologies, A11031) and Alexa Fluor 647 donkey anti-rabbit (1:100; Life Technologies, A31573). For wing imaginal discs, P-cad and pSrc staining were performed by dissecting third-instar larvae in phosphate buffer at pH 7 (0.1 M Na_2_HPO_4_, 0.1 M NaH_2_PO_4_ at a 72:28 ratio). Discs were then fixed in 4% formaldehyde in PEM (0.1 M PIPES pH 7, 0.2 mM MgSO4, 1 mM EGTA) for 15-30 min, rinsed in phosphate buffer 0.2% Triton X-100 for 15 min and incubated with rabbit anti-P-cad (1:50; Cell Signaling Technology, 2130S, lot #2), rabbit anti-pSrc (pY419) (1:10; Invitrogen, 44-660G, lot #2253011; see Fernández et al., 2014 for antibody specificity in *Drosophila* epithelia), rat anti-Zfh2 (Tran et al., 2010) or rabbit anti-pH3 (1:200; Cell Signaling Technology, 9701, lot #3) overnight at 4°C. Discs were then rinsed three times for 10 min in phosphate buffer 0.2% Triton X-100, incubated for 1 h at room temperature (RT) with secondary antibodies (Jackson ImmunoResearch) in phosphate buffer 0.2% Triton X-100 supplemented with 10% horse serum and rinsed three more times for 10 min before being mounted in Vectashield (Vector Laboratories, H-1000). For Phalloidin staining, discs were dissected and fixed as described above and incubated for 1 h at RT with Rhodamine-conjugated Phalloidin (Sigma-Aldrich, P-1951) at 0.3 mM in phosphate buffer 0.2% Triton X-100 supplemented with 10% horse serum. Discs were then rinsed three more times for 10 min before being mounted in Vectashield (Vector Laboratories, H-1000). For salivary glands, third-instar larvae were dissected on ice in phosphate buffer and fixed in 4% formaldehyde in PEM, according to standard protocol. Samples were incubated with rabbit anti-P-cad (1:50; Cell Signaling Technology, 2130S, lot #2) or mouse anti-GFP (1:500; DSHB, GFP-12A6) in phosphate buffer 0.2% Triton X-100, supplemented with 10% FBS, overnight at 4°C. Alexa Fluor anti-rabbit and anti-mouse secondary antibodies were diluted in phosphate buffer 0.2% Triton X-100, supplemented with 10% FBS (1:1000; Invitrogen). 4′,6-Diamidino-2-phenylindole (DAPI) was used to counterstain the nuclei, and samples were mounted in 50% glycerol/PBS. Fluorescence images were obtained on LSM 510 Zeiss or Leica SP5 Live confocal microscopes using 10× dry, 40× water or 63× glycerol objectives or on a Leica Scanning Confocal SP8 TCS SP8 microscope with HC FLUOTAR L 25× objective.

For staining MCF10A-ER-Src cells, 75,000 cells were seeded on poly-L-lysine-coated coverslips for at least 16 h before being treated with 1 µM 4OH-TAM or an identical volume of EtOH for the indicated times. Cells were then rinsed with 1× PBS and fixed with 4% paraformaldehyde in 1× PBS at pH 7 for 10 min at RT. Cells were then permeabilized with Tris-buffered saline (TBS) with 0.1% Triton X-100 (TBS-T) for 2 min at RT and blocked with blocking buffer (10 mM MES pH 6.1, 150 mM NaCl_2_, 5 mM EGTA pH 6.8, 5 mM MgCl_2_, 5 mM glucose, 2% FBS and 1% BSA) for 1 h at RT. Mouse anti-MRTF-A (G-8) monoclonal antibody (1:50; Santa Cruz Biotechnology, sc-390324, lot #L1719), rabbit anti-P-cad antibody (1:50; Cell Signaling Technology, 2130S, lot #2) or mouse anti-p120^ctn^ monoclonal antibody (1:100; BD Transduction Laboratories, 610133, lot #5338751) were incubated overnight at 4°C in blocking buffer. Coverslips were washed with 1× PBS three times for 5 min at RT and incubated with donkey anti-mouse IgG Alexa Fluor 488 secondary antibody (1:200; Jackson ImmunoResearch, 715-095-150) or Cy5-conjugated donkey anti-mouse (1:200; Jackson ImmunoResearch, 715-175-020) or TRITC-conjugated donkey anti-rabbit (1:200; Jackson ImmunoResearch, 711-095-152) and Rhodamine-conjugated Phalloidin (Sigma-Aldrich, P-1951) at 0.3 mM in blocking buffer for 1 h at RT in dark. After three washes in 1× PBS, cells were stained with DAPI (1:500; Sigma-Aldrich, D9542) for 5 min at RT, washed again with 1× PBS and mounted in Vectashield. Fluorescence images were obtained on a Leica SP5 confocal coupled to a Leica DMI6000, using the 63×1.4 HCX PL APO CS oil immersion objective. Image processing was performed using Imaris software.

### Immunoblotting analysis and quantification

Protein extracts from wing imaginal discs were obtained by dissecting six discs in phosphate buffer at pH 7 (0.1 M Na_2_HPO_4_, 0.1 M NaH_2_PO_4_ at a 72:28 ratio). Discs in 28 μl PBS were lysed by adding 10 μl 5× Laemmli buffer, 7 μl 7× proteinase inhibitor (Roche, 04693159001) and 5 μl 10× phosphatase inhibitor (Roche, 04906837001). Protein extracts from MCF10A-ER-Src cells were obtained by incubating cell pellets in Lysis Buffer SDS-Free, composed of 0.05 M Tris-HCl pH 7.5 (VWR, 33621.260), 0.15 M NaCl (Sigma-Aldrich, 31434), 0.001 M EDTA pH 8 (Sigma-Aldrich, E5134), 0.001 M EGTA pH 7 (Sigma-Aldrich, E3889), 1% Triton X-100 (Sigma-Aldrich, T8787), and containing 1% protease inhibitors and 1% phosphatase inhibitors on ice for 30 min. Lysis products were centrifuged at 4°C for 30 min at 19,283 ***g***, and protein was quantified using the Bradford method. Laemmli buffer was added to a final concentration of 1×. Protein extracts from wing discs and MCF10A-ER-Src cells were then boiled for 5 min at 95°C and centrifuged for 10 min at 5900 ***g*** before being resolved by SDS-PAGE electrophoresis and transferred to a 0.45 µM PVDF blotting membrane (Amersham, 10600023), previously activated for 5 min in methanol. Membranes were blocked with 5% milk in TBS with 0.1% Tween 20, cut in half at ∼50 kDa to separate proteins migrating at higher and lower molecular masses and incubated with mouse anti-P-cad (1:2500; BD Biosciences, 610228, lot #6197932; see Vieira et al., 2012 for antibody specificity), mouse anti-MRTF-A (1:1000; Santa Cruz Biotechnology, sc-390324, lot #L1719), mouse anti-HSC70 (1:8000; Santa Cruz Biotechnology, sc-7298, lot #I2520), rabbit anti-H3 (1:3000; Cell Signaling Technology, 9715, lot #20) or mouse anti-actin (1:1000; Cytoskeleton, Inc., AAN02-S, lot #113) diluted in TBS with 0.1% Tween 20 supplemented with 5% non-fat milk or 3% BSA. Secondary antibodies used were horseradish peroxidase (HRP)-conjugated AffiniPure donkey anti-mouse IgG (1:5000; Jackson ImmunoResearch, 715-035-150), HRP-conjugated AffiniPure donkey anti-rabbit igG (1:5000; Jackson ImmunoResearch, 711-035-152), IRDye^®^ 680RD-conjugated donkey anti-mouse IgG (1:20,000; LI-COR Bioscences, 926-68072) and IRDye^®^ 800CW-conjugated donkey anti-rabbit IgG (1:20,000; LI-COR Bioscences, 926-32213). Detection was performed using Immobilon Western Chemiluminescent HRP substrate (Millipore, P90719), and visualization was performed using a ChemiDoc (Bio-Rad) or Odyssey^®^ M (LI-COR Biosciences) imaging system.

### Flow cytometry

MCF10A-ER-Src cells treated with 4OH-TAM or EtOH for the indicated time were washed twice with Dulbecco's phosphate buffered saline (DPBS; Biowest, L0615). Cells were then harvested with Versene Solution (Gibco, 15040-033), washed with DPBS supplemented with 0.5% FBS (stain buffer), centrifuged at 270 ***g*** for 5 min and resuspended in stain buffer. Then, 500,000 cells were stained for 20 min at 4°C in the dark with APC-conjugated anti-P-cad antibody (1:10; R&D Systems, FAB861A, lot #lWO02117051) and a Live/Dead™ Fixable Violet Dead Cell Stain Kit (1:1000; Invitrogen, L34955) in 100 µl stain buffer. Cells were then washed twice with 1 ml cold stain buffer followed by centrifugation at 270 ***g*** for 5 min at 4°C. Cells were resuspended in 500 µl cold stain buffer, passed through a 35 µm nylon cell strainer (Corning, 352235) to remove clumps and analyzed on a FACS Canto II (BD Biosciences) equipped with the acquisition software BD FACSDiva (BD Biosciences). Data analysis was performed using FlowJo software version 10.5.3 (TreeStar, Inc.), and only viable (Live/Dead negative) single cells without debris were included in the analysis.

### Transfections and dual Luciferase Renilla reporter assay

First, 150,000 cells resuspended in 2 ml CGM were plated in six-well plates. After 12-24 h, the cells were transfected with 0.720 µg p3D.A-Luc and 1.44 µg pRL-TK plasmids (Posern et al., 2002) with or without 50 pmol siCTR (Qiagen, 1027310) or siCDH3 (Qiagen, 1027416, GeneGlobe ID: SI02663941) using Lipofectamine 2000 (Invitrogen, 11668-019), according to the manufacturer's instructions. After 12 h, transfected cells were incubated for another 12 h in RGM. Cells were then treated with EtOH or TAM with or without LatA or CCG-203971 for 6 h in RGM. After trypsinization with TrypLE™ Express (Gibco, 12604-021), cell pellets were resuspended in 250 µl 1× passive lysis buffer (Promega, E1941) and kept overnight at −20°C. Luciferase assay was performed using a Dual-Luciferase Reporter Assay system (Promega, PROME19600010) according to the manufacturer's instructions. Briefly, 100 µl Luciferase assay reagent II (LARII) was added to 80 µl lysed samples in a 96-well plate. Firefly luminescence was detected using Synergy Mx (BioTek). Then, 100 µl 1× Stop and Glo solution (Promega, PROME19600010) was added to detect Renilla luminescence. Each sample was evaluated in triplicates.

### Trypan Blue cell viability assay

Cells were washed with DPBS and trypsinized using TrypLE™ Express for 15 min in a 5% CO_2_ humidified incubator at 37°C. After centrifugation at 200 ***g*** for 5 min, cell pellets were resuspended in 20 µl RGM. For each experimental condition, cells in 5 µl cell suspension in the same volume of Trypan Blue (Lonza, LONZ17-942E) were counted in a Neubauer chamber.

### Cell cycle profile

First, 265,000 cells in 4 ml CGM were plated in a T25 flask for 12-24 h. To test the effect of knocking down *P-cad* on the proliferation of EtOH- or TAM-treated cells, cells were transfected with siCtr (Qiagen, 1027310) or *siP-cad* (Qiagen, 1027416, GeneGlobe ID: SI02663941) using Lipofectamine 2000 (Invitrogen, 11668-019), according to the manufacturers’ instructions. After 12 h, transfected cells were incubated for 24 h in RGM and treated with EtOH or TAM for 12 h or incubated for another 12 h in RGM and treated with EtOH or TAM for 24 h, before trypsinization. To test the effect of inhibiting MRTF-A nuclear translocation on the proliferation of EtOH- or TAM-treated cells, cells were washed three times with DMEM/F12 and incubated for 12 h in RGM. Cells were then treated with EtOH or TAM in the presence or absence of 40 µM CCG-203971 for 12 or 24 h in RGM. Cells were then trypsinized with TrypLE™ Express and collected in 5 ml round-bottom polystyrene tubes (Corning, 352235). After centrifugation for 5 min at 180 ***g*** at 4°C, cells were resuspended in 1 ml FACS Buffer 1, composed of 2% heat-inactivated FBS (Biowest, S181BH) in 1× DPBS, centrifuged again at 4°C for 5 min at 180 ***g*** and resuspended in 500 µl FACS Buffer 1. Cells were then fixed with 1.5 ml 70% EtOH, added drop by drop, while being gently vortexed, followed by a 30 min incubation at 4°C. After 5 min centrifugation at 720 ***g*** at 4°C, cells were resuspended in 3 ml 1× DPBS, incubated for 30 min on ice, pelleted again by centrifugation for 5 min at 720 ***g*** at 4°C and resuspended in 300 µl FACS Buffer 2, composed of 100 µg/ml RNAse A (Qiagen, 19101) and 20 µg/ml propidium iodide (Sigma-Aldrich, P4170) in 1× DPBS. Samples were incubated for 30 min in a 37°C water bath in the dark. Flow cytometry was performed at low flow rate using a BD Accuri C6 Flow Cytometer (Becton-Dickinson). The cell cycle profile was analyzed using FlowJo 10.7.1 software (Tree Star, Inc.), Cell Cycle platform, using the Watson Model.

### Invasion assay in collagen

First, 20,000 cells in 200 µl of CGM were plated in a 96-well plate, previously coated with 30 µl 0.7% agarose (Lonza, 50004). The cells were allowed to form spheroids for 24 h. The next day, medium was replaced by 60 µl 5 mg/ml collagen type I (VWR, 734-1085) in 1 M NaOH and 1× DMEM/F12. After 1 h, spheroids were treated with 4 µM TAM or the same volume of EtOH in the presence or absence of 160 µM CCG-203971 and incubated in a 5% CO_2_ humidified incubator at 37°C for 36 h. Brightfield pictures were taken using a 5× objective on a brightfield microscope.

### Mammosphere assay

First, 150,000 cells in 2 ml CGM were plated in a six-well plate. After 12-24 h, cells were treated with EtOH or TAM in the presence or absence of 40 µM CCG-203971 for 6 or 36 h in RGM. Cells were trypsinized with Versene, resuspended in 1 ml 1× DPBS and passed three times through a 25-gauge needle to obtain single-cell suspensions. Then, 10,000 cells were plated in six-well plates previously coated with Poly(2-hydrocyethyl methacrylate) (PolyHEMA) and incubated in 2 ml mammosphere medium composed of 1:1 DMEM/F12, 20 ng/ml human EGF, 40 µg/ml insulin, 500 ng/ml hydrocortisone, 1% penincilin/streptomycin (Merck, 15070-063) and 1:1 Supplement B-27 Minus Vitamin A (Gibco, 12587-010), filtered with a 0.45 µm filter (VWR, 514-4127). Six days after incubation in a 5% CO_2_ humidified incubator at 37°C, brightfield pictures were acquired using a 5× objective on a brightfield microscope. Each condition was evaluated in triplicates.

### Quantifications

The National Institutes of Health (NIH) ImageJ program was used to quantify wing disc area. Except for *nub>GFP* control wings, comparisons were performed on wings containing the same number of UAS transgenes. UAS-*GFP* or UAS-*mCherry* was used for normalization when needed. To quantify the total adult wing area, each wing excluding the costal cell and alar lobe was outlined using the freehand section tool and measured using the Area function, which evaluates size in square pixels. To quantify adult wing circularity, each wing, excluding the costal cell and alar lobe, was outlined using the freehand section tool and measured using the circularity tool [4π×(area)/(perimeter)^2^] within the Shape descriptors function, which evaluates how close to a perfect circle shapes are, with a value of 1 indicating a perfect circle. To quantify adult wing A/P lengths, a line was drawn between the tip of longitudinal vein 0 and the tip of longitudinal vein 3 for each disc using the straight-line section tool and measured using the length tool within the Area function, which evaluates length in square pixels. The perimeter of the *Drosophila* adult eyes was also measured using ImageJ.

To quantify the percentage of pH3 signal in the dorsal blade domain over the total blade domain, we extracted automatically the number of pH3 signals exclusively in the domain that do not express Zfh2 or that do not express Zfh2 but express GFP using the Spot function in the Imaris software. The numbers of pH3 spots in each domain were then normalized to their respective area by outlining the Zfh2-negative or the Zfh2-negative, GFP-positive wing disc area using the freehand section tool from ImageJ and by measuring their size in square pixels using the Area function. To evaluate nuclear accumulation of Mrtf.3XGFP, we used surface segmentation tools from Imaris software to define nuclei area, on 3D projections of salivary gland images. The intensity of Mrtf.3XGFP signals within the defined nuclei was extracted automatically. ImageJ was used to evaluate MRTF-A nuclear accumulation. Nuclear MRTF-A fluorescence intensity was measured as mean gray intensity value for each nucleus, defined using the DAPI channel. Fold changes in nuclear MRTF-A were calculated after normalization to MRTF-A nuclear intensity in EtOH-treated cells. Western blot quantifications were performed using Image Lab software (Bio-Rad). Quantifications of the levels of P-cad in wing discs correspond to the sum of the bands at higher (P-cad pro-peptide) and lower (mature P-cad protein) molecular masses. For each experimental condition, P-cad levels were evaluated by normalizing the P-cad signal with that of the corresponding H3 signal from the same gel. Relative Luciferase activity was quantified by normalizing Firefly Luciferase activity to respective Renilla Luciferase activity. Western blot quantification was performed using Image Lab software for images acquired using ChemiDoc or with Empiria software (LI-COR Biosciences) for images acquired using the Odyssey^®^ M imaging system. Band intensities were determined in the Analysis table function. To obtain the final quantification, the intensity levels of each band were normalized to those of its respective control. Spheroid's circularity was quantified using ImageJ. Each spheroid was outlined using the freehand selection tool and measured using the shape descriptor tool. Mammospheres with ≥60 µm diameter were counted using a 5× objective on a brightfield microscope. To calculate mammosphere-forming efficiency, the number of mammospheres counted was divided by the number of cells plated (10,000 cells). GraphPad Prism (9.0) was used for data presentation and statistical analysis.

### Methodology and statistics

No statistical methods were used to predetermine sample size. To select adult females used for quantification of wing size and circularity, only vials in which pupae did not reach the plug were considered. All other vials were discarded. Damaged wings due to handling were excluded for quantifications. To quantify total actin levels by western blotting in wing disc extracts, one biological replicate was excluded because no signal could be detected in control discs expressing GFP (Fig. 3B). To quantify P-cad levels by western blotting in wing disc extracts knocked down for *dia*, one biological replicate was excluded because the signal of the two P-cad bands was unequal (Fig. 3E). To evaluate the percentage of S-phase cells in MCF10A-ER-Src cells transfected with *siCtr* or *siP-cad* and treated with EtOH or TAM for 12 h, one experiment was excluded (Fig. 4H), as the number of S-phase cells in TAM-treated cells was more than twice that in other experiments. Yet, in this experiment, knocking down P-cad reduced by almost twofold the number of TAM-treated cells in S-phase. We excluded two experiments to determine the effect of preventing F-actin polymerization on SRF-Luc activity (Fig. 6C). One experiment showed more than twofold variabilities in Renilla luminescence between experimental conditions. In another experiment, TAM-treated cells did not show higher SRF-Luc activity compared to those treated with EtOH in the absence of LatA. To evaluate the effect of blocking MRTF-A nuclear translocation on the ability of MCF10A-ER-Src cells to sustain proliferation (Fig. 7B), one biological replicate was excluded, as TAM-treated cells did not show a higher percentage of cells in S-phase, compared to those treated with EtOH in the absence of CCG-203971. For other experiments, all experiments were considered. All analyses were performed using GraphPad Prism 6.0 software. Normal distribution of the data was tested using a Shapiro–Wilk test. For statistical comparison of two independent groups (Fig. 2D, Fig. 3B,D,E, Fig. 4C, Fig. 6B and Fig. 7E; Fig. S2 and Fig. S3D), unpaired two-tailed Student's *t*-test was used. For statistical comparison between more than two groups (Fig. 1F,G,K,L, Fig. 2A,B, Fig. 3C, Fig. 4B,C,E,G,H, Fig. 5A,B,D-F, Fig. 6C, Fig. 7A-D; Fig. S1C, Figs S5-S8), one-way ANOVA with Tukey's multiple comparison was used.

## Supplementary Material

10.1242/dmm.049652_sup1 Supplementary information
