# Supplementary material for: Activation of an actin signaling pathway in pre-malignant mammary epithelial cells by P-cadherin is essential for transformation
Source: Dis Model Mech. 2023 Feb 21;16(2):dmm049652. doi: 10.1242/dmm.049652 (PMC9983776; doi:10.1242/dmm.049652)
Supplement: Supplementary information [file dmm-16-049652-s1.pdf]

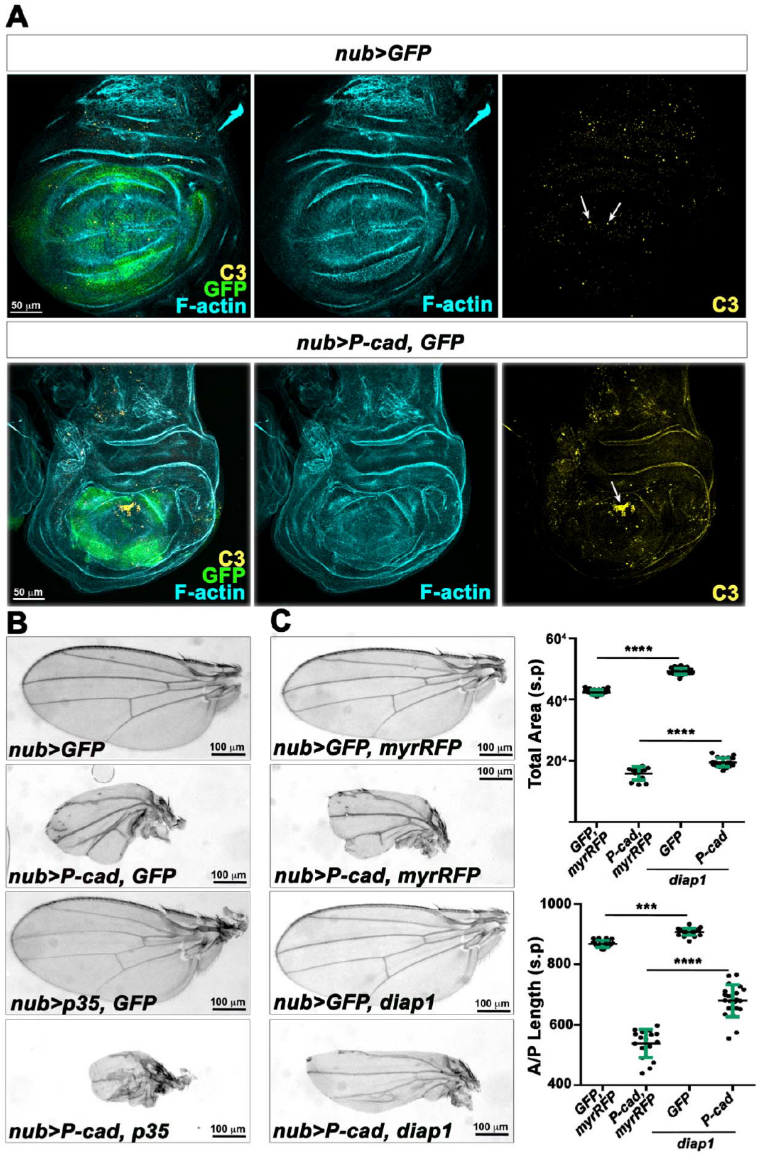

Fig. S1. Tissue loss by apoptosis could contribute to the reduced size of *P-cad*-expressing wings. (A) Standard confocal sections of third instar wing imaginal discs expressing UAS-*mCD8-GFP* (green) or UAS-*mCD8-GFP* (green) and UAS-*P-cad* under *nub*-Gal4 control and stained with Phalloidin (cyan) to mark F-actin and anti-activated Caspase 3 (C3) (yellow). White arrows indicate apoptotic cells. Scale bar represent 50 µm. The staining was replicated twice **(B)** Adult wings in which *nub*-Gal4 drives UAS-*mCD8-GFP* alone or UAS-*mCD8-GFP* and UAS-*P-cad* or UAS-*mCD8-GFP* and UAS-*p35* or UAS-*p35* and UAS-*P-cad*. Wings were from one cross **(C)** (Left panels) Adult wings in which *nub*-Gal4 drives UAS-*mCD8-GFP* and UAS-*myr-RFP* or UAS-*myr-RFP* and UAS-*P-cad* or UAS-*mCD8-GFP* and UAS-*Diap1* or UAS-*P-cad and* UAS-*Diap1*. Scale bar represents 100 µm. (Right panels) Quantifications from one biological replicates of the total area (top) or anterior-posterior length (bottom) of adult wings in which *nub*-Gal4 drives UAS-*mCD8-GFP* and UAS-*myr-RFP* (n=25) or UAS-*myr-RFP* and UAS-*P-cad* (n=13) or UAS-*mCD8-GFP* and UAS-*Diap1* (n=25) or UAS-*P-cad and* UAS-*Diap1* (n=21). s.p. indicates square pixels. Error bars indicate SD. *** indicates p<0.001; **** indicates p<0.0001**.** Statistical significance was calculated using one-way ANOVA with Tukey´s multiple comparisons tests.

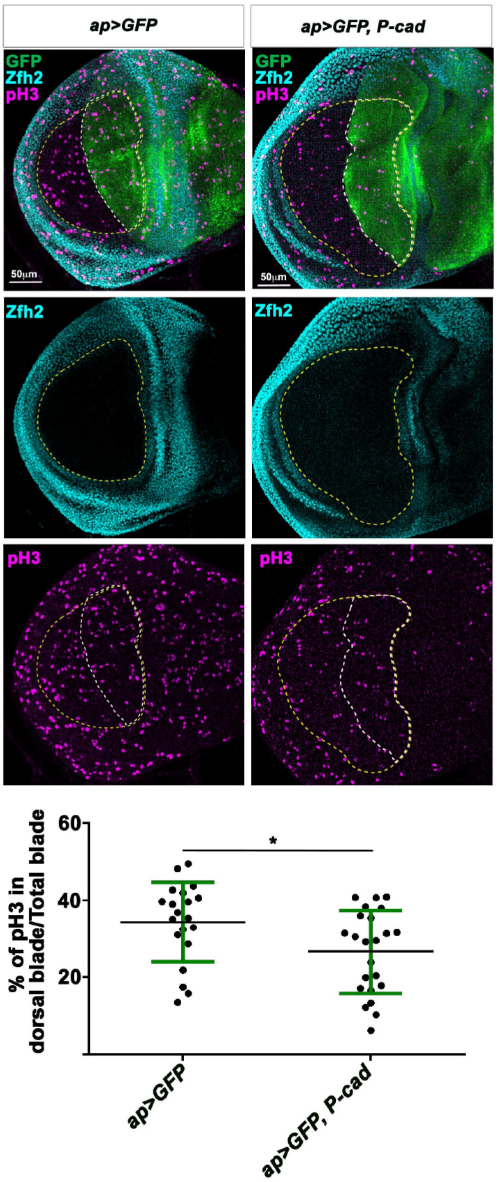

Fig. S2. P-cad restricts cell proliferation in the wing blade. (Top panels) Standard confocal sections of third instar wing imaginal discs expressing UAS-*mCD8-GFP* (green) or UAS-*mCD8-GFP* (green) and UAS-*P-cad* under *ap*-Gal4 control and stained with anti-Zfh2 (cyan) to mark the hinge domain and anti-phosphorylated Histone 3 (pH3) (magenta). Yellow dashed lines delimitate the wing blade cells that do not express Zfh2. White dashed lines delimitate the dorsal blade domain expressing GFP but not Zfh2. Scale bar correspond to 50 µm. (Lower panel) Quantification from three biological replicates of the percentage (%) of pH3-positive cells in the dorsal blade over the total blade domains for wing discs in which *ap*-Gal4 drives UAS-*mCD8-GFP* (n=21) or UAS-*mCD8-GFP* (green) and UAS-*P-cad* (n=23), normalized to the area of the dorsal blade and total blade, respectively. Error bars indicate SD. * indicates $p<0.05$. Statistical significance was calculated using unpaired two-tailed Student´s t-test.

Disease Models & Mechanisms • Supplementary information

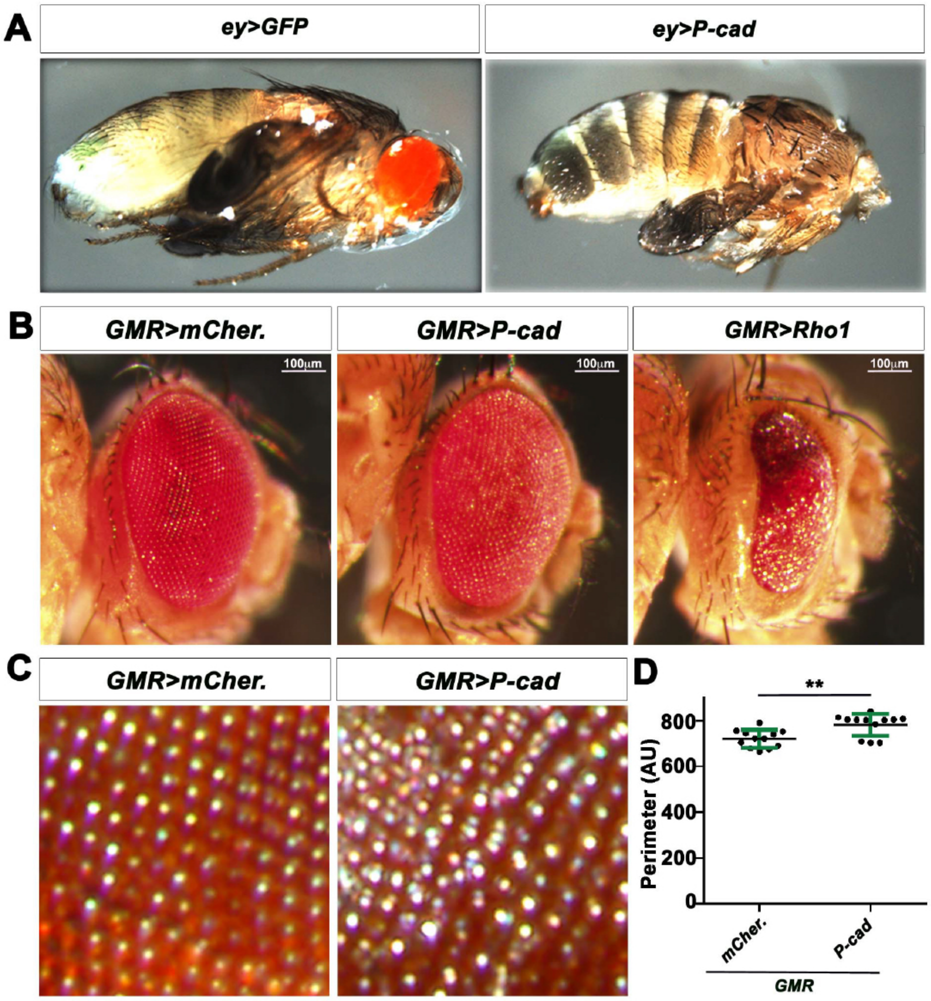

Fig. S3. P-cad induces major defects when expressed in undifferentiated/progenitor cells of the eye imaginal discs, while has weaker effects in epithelial cells undergoing differentiation posterior to the morphogenetic furrow. (A) Pupae in which *ey*-Gal4 drives UAS-*mCD8-GFP* or UAS-*P-cad.* Crosses were replicated twice. **(B)** Adult eyes in which *GMR*-Gal4 drives UAS-*mCherry* or UAS-*P-cad* or UAS-*Rho1*. Crosses were replicated twice **(C)** Magnification of adult retina in which *GMR*-Gal4 drives UAS-*mCherry* or UAS-*P-cad.* **(D)** Quantification from one biological replicate of the perimeter of adult eyes in which *GMR*-Gal4 drives UAS-*mCherry* (n=12) or UAS-*P-cad* (n=12). AU indicates arbitrary unit. Error bars indicate SD. ** indicates p<0.01. Statistical significance was calculated using unpaired two-tailed Student´s t-test.

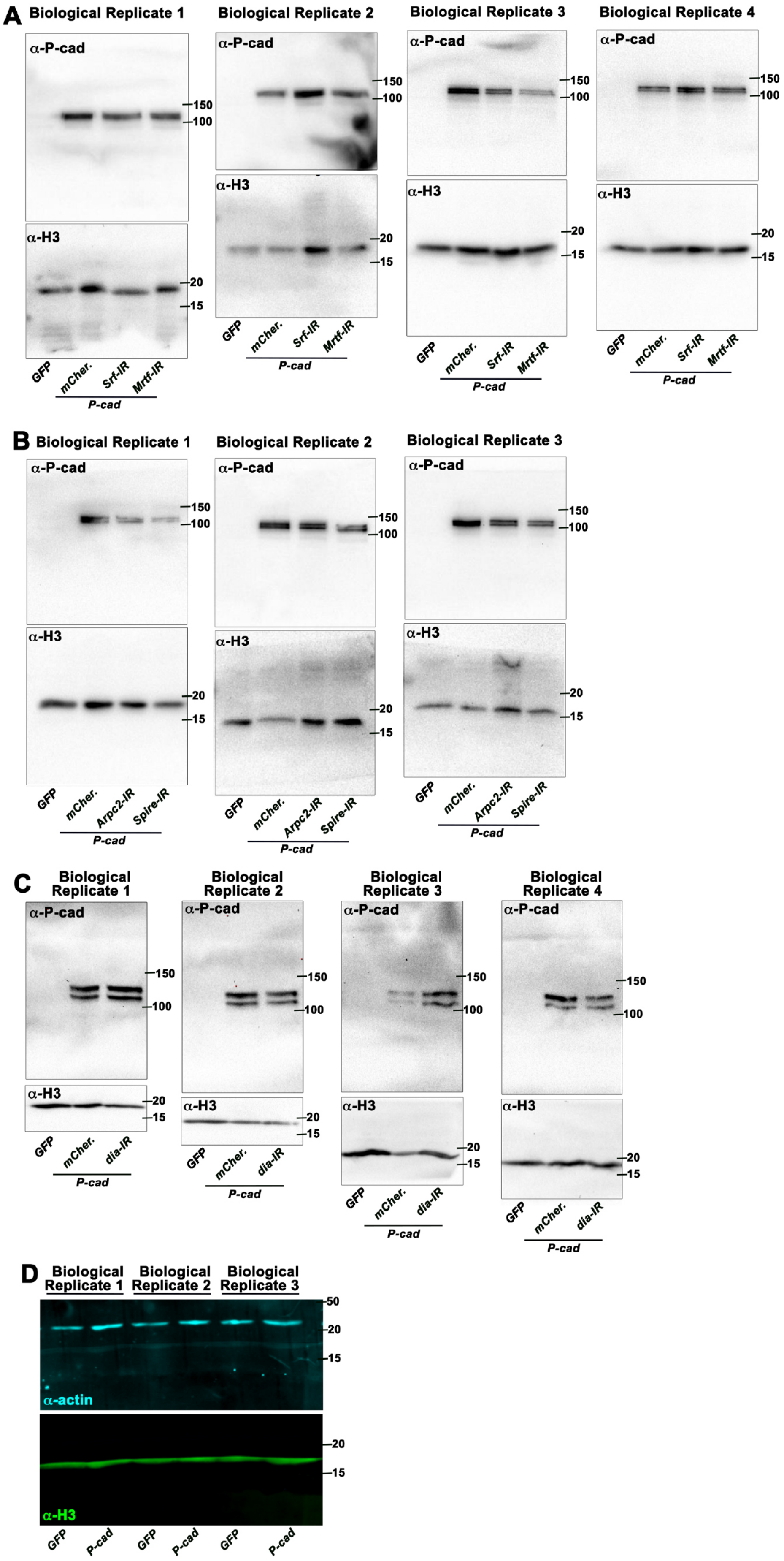

Disease Models & Mechanisms • Supplementary information

Fig. S4. P-cad levels are not affected in wing discs knocked down for *Mrft, Srf* or *dia* but are reduced in those knocking down for *Arpc2* or spire. (A) Four biological replicates of western blots on protein extracts from wing imaginal discs expressing UAS-*CD8-GFP* or UAS-*P-cad* and UAS-*mCherry* or UAS-*P-cad* and UAS-*Mrtf-IR* or UAS-*P-cad* and UAS-*Srf-IR* under *nub*-Gal4 control, blotted with anti-P-cad and anti-Histone 3 (α−H3) used as loading control. **(B)** Three biological replicates of western blots on protein extracts from wing imaginal discs expressing UAS-*CD8-GFP* or UAS-*P-cad* and UAS-*mCherry* or UAS-*P-cad* and UAS-*Arpc2-IR* or UAS-*P-cad* and UAS-*spire-IR* under *nub*-Gal4 control, blotted with anti-P-cad and anti-Histone 3 (α−H3) used as loading control. **(C)** Four biological replicates of western blots on protein extracts from wing imaginal discs expressing UAS-*CD8-GFP* or UAS-*P-cad* and UAS-*mCherry* or UAS-*P-cad* and UAS-*dia-IR* under *nub*-Gal4 control, blotted with anti-P-cad and anti-Histone 3 (α−H3) used as loading control. (D) Three biological replicates of western blots on protein extracts from wing imaginal discs expressing UAS-*CD8-GFP* or UAS-*P-cad* under *ap*-Gal4 control, blotted with anti-actin and anti-Histone 3 (α−H3) used as loading control.

Disease Models & Mechanisms • Supplementary information

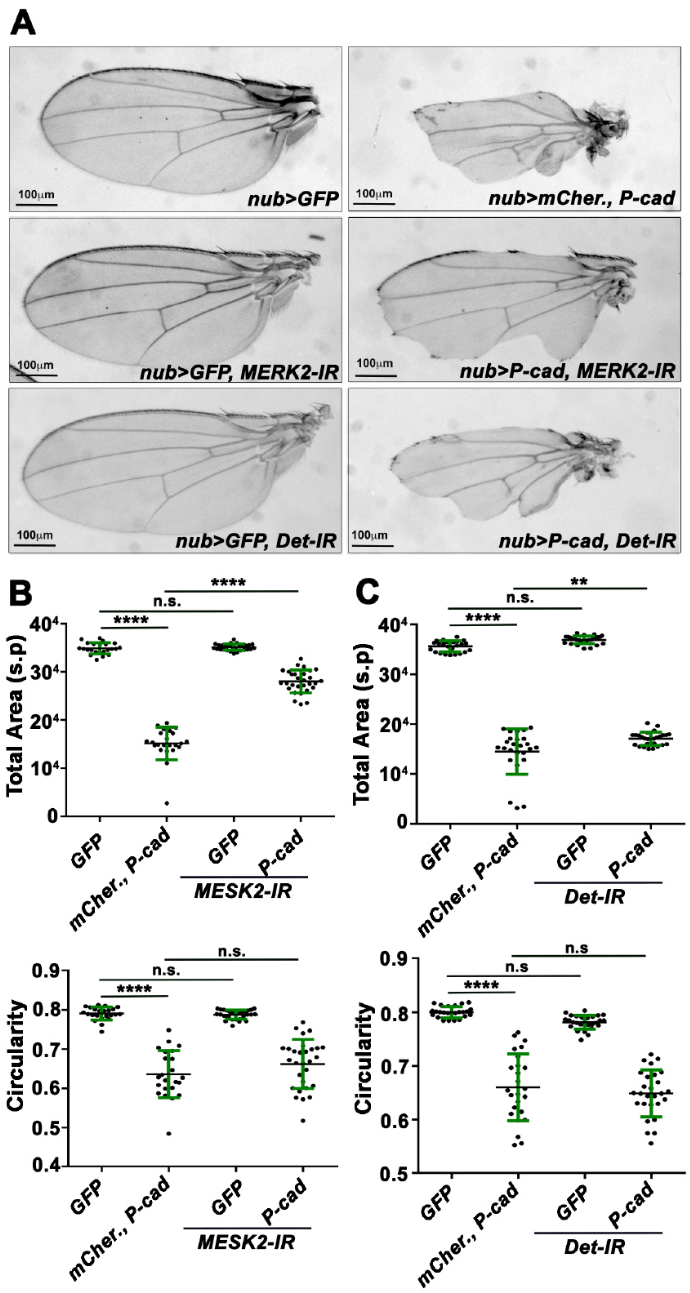

Fig. S5. Knocking down the Mrtf-Srf transcriptional targets MESK2 or Det restores the size of *P-cad*-expressing wings. (A) Adult wings in which *nub*-Gal4 drives UAS-*mCD8-GFP* or UAS-*P-cad* and UAS-*mCherry* or UAS-*MESK2-IR*^{JF03312} and UAS-*mCD8-GFP* or UAS-*MESK2-IR*^{JF03312} and UAS-*P-cad* or UAS-*Det-IR*^{GL00572} and UAS-*mCD8-GFP* or UAS-*Det-IR*^{GL00572} and UAS-*P-cad.* **(B-C)** Quantification of the total area and circularity of wings in which *nub*-Gal4 drives **(B)** UAS-*mCD8-GFP* (n=23) or UAS-*P-cad* and UAS-*mCherry* (n=23) or UAS-*MESK2-IR*^{JF03312} and UAS-*mCD8-GFP* (n=28) or UAS-*MESK2-IR*^{JF03312} and UAS-*P-cad* (n=29) or **(C)** UAS-*mCD8-GFP* (n=25) or UAS-*P-cad* and UAS-*mCherry* (n=25) or UAS-*Det-IR*^{GL00572} and UAS-*mCD8-GFP* (n=27) or UAS-*Det-IR*^{GL00572} and UAS-*P-cad* (n=27). s.p indicates square pixels. Quantification of control *nub>GFP* and *nub>mCher., P-cad* are from three biological replicates. Other quantifications are from one biological replicate. Error bars indicate SD; n.s. indicate non-significant; ** indicates P<0.01; **** indicates P<0.0001**.** Statistical significance was calculated using one-way ANOVA with Tukey´s multiple comparisons tests.

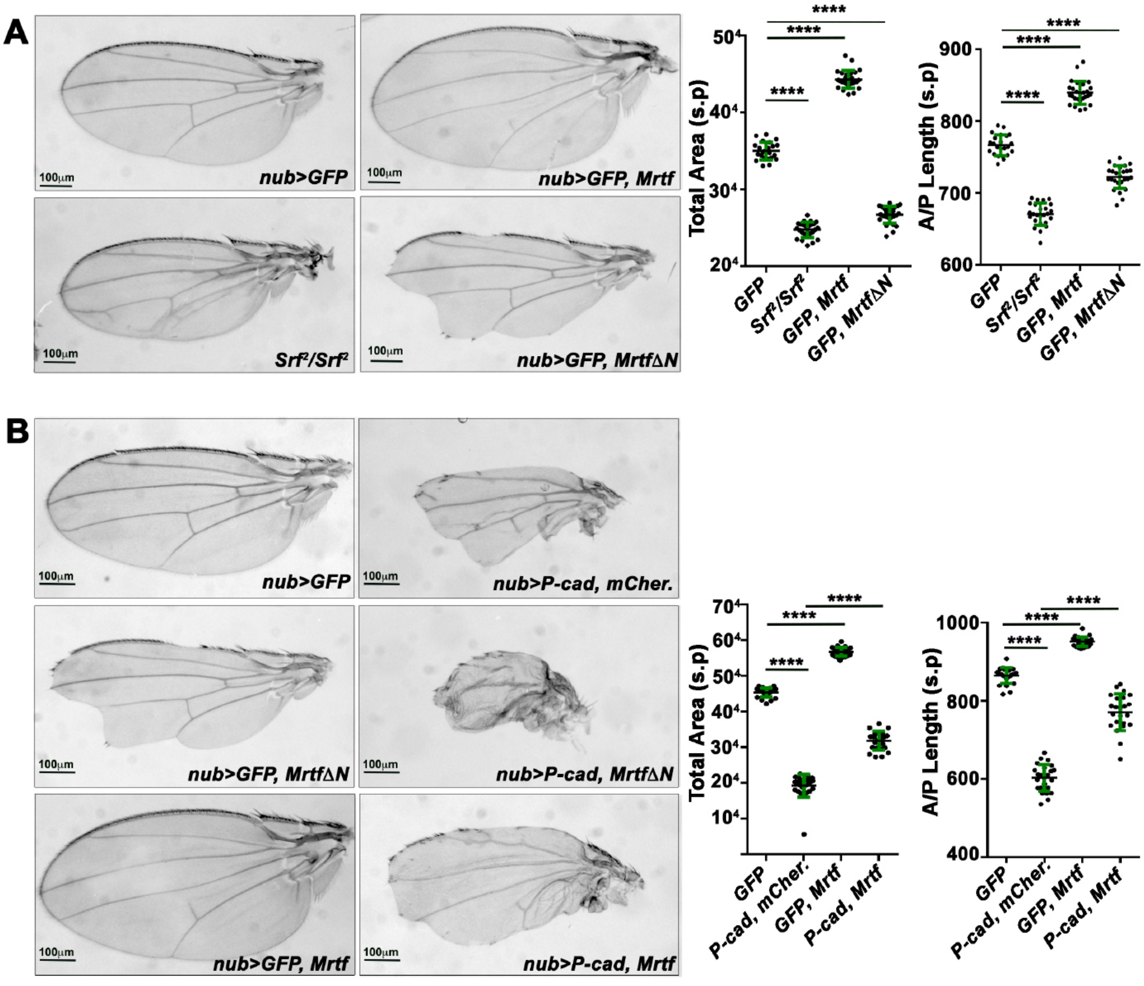

Fig. S6. Expressing a constitutive active form of *Mrtf* phenocopies the *P-cad*-expressing wing phenotype. (A) (Left panels) Adult wings in which *nub*-Gal4 drives UAS-*mCD8-GFP* or UAS-*Mrtf* or UAS-*MrtfΔN* or homozygous mutant for the bs^2 allele (Srf^2/Srf^2). Scale bar represent 100 µm. (Right panels) Quantifications of the total area or anterior-posterior (A/P) length of adult wings in which *nub*-Gal4 drives UAS-*mCD8-GFP* (n=23) or UAS-*Mrtf* (n=27) or UAS-*MrtfΔN* (n=26) or homozygous mutant for the bs^2 allele (Srf^2/Stf^2; n=25). **(B)** (Left panels) Adult wings in which *nub*-Gal4 drives UAS-*mCD8-GFP* or UAS-*P-cad* and UAS-*mCherry* or UAS-*Mrtf* and UAS-*mCD8-GFP* or UAS-*Mrtf* and UAS-*P-cad* or UAS-*MrtfΔN* and UAS-*mCD8-GFP* or UAS-*MrtfΔN* and UAS-*P-cad.* (Right panels) Quantifications from one biological replicate of the total area or anterior-posterior (A/P) length of adult wings in which *nub*-Gal4 drives UAS-*mCD8-GFP* (n=25) or UAS-*P-cad* and UAS-*mCherry* (n=25) or UAS-*Mrtf* and UAS-*mCD8-GFP* (n=25) or UAS-*Mrtf* and UAS-*P-cad* (n=25). s.p indicates square pixels. Quantification of control *nub>GFP* and *nub>mCher., P-cad* are from three biological replicates. Other quantifications are from one biological replicate. Error bars indicate SD; **** indicates P<0.0001**.** Statistical significance was calculated using one-way ANOVA with Tukey´s multiple comparisons tests.

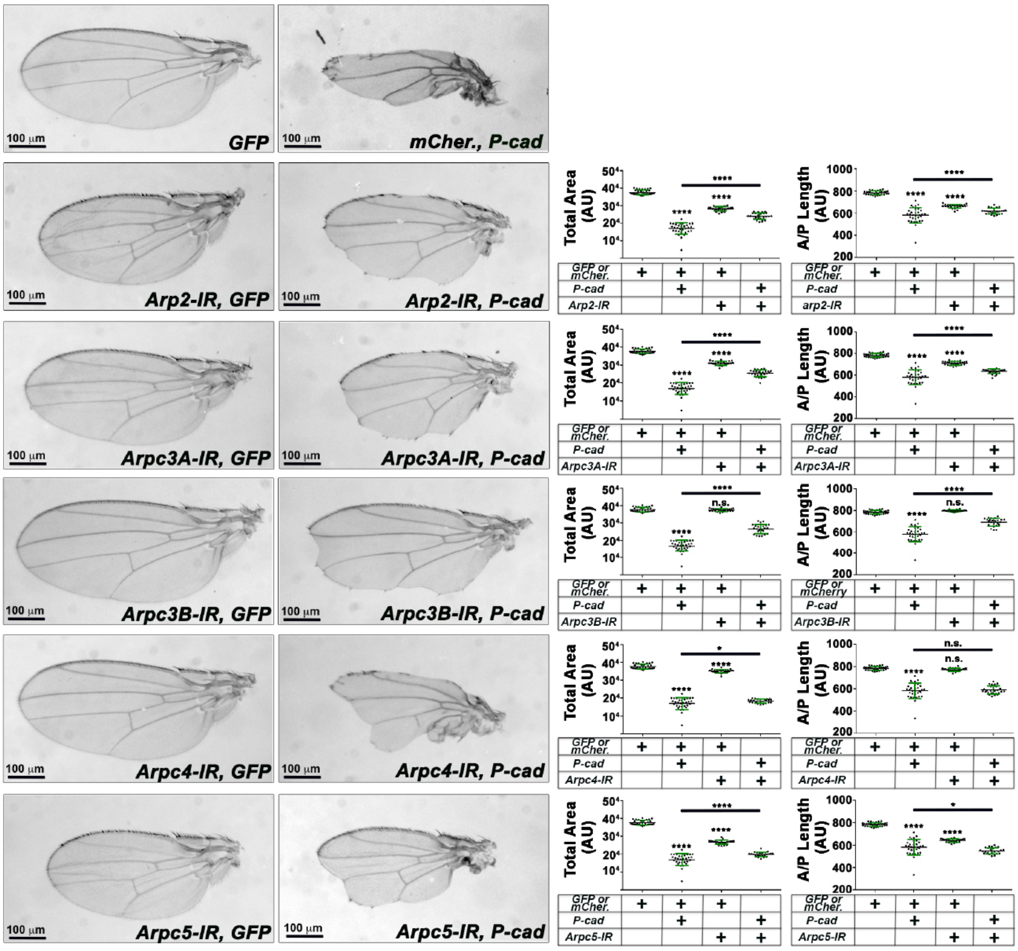

Fig. S7. Knocking down subunits of the Arp2/3 complex suppresses the *P-cad*-expressing wing phenotype. (Left panels) Adult wings in which *nub*-Gal4 drives UAS-*mCD8-GFP* or UAS-*P-cad* and UAS-*mCherry* or UAS-*Arp2-IR*^{JF02785} and UAS-*mCD8-GFP* or UAS-*Arp2-IR*^{JF02785} and UAS-*P-cad* or UAS-*Arpc3A-IR*^{JF02370} and UAS-*mCD8-GFP* or UAS-*Arpc3A-IR*^{JF02370} and UAS-*P-cad* or UAS-*Arpc3B-IR*^{JF02679} and UAS-*mCD8-GFP* or UAS-*Arpc3B-IR*^{JF02679} and UAS-*P-cad* or UAS-*Arpc4-IR*^{JF01683} and UAS-*mCD8-GFP* or UAS-*Arpc4-IR*^{JF01683} and UAS-*P-cad* or UAS-*Arpc5-IR*^{JF03147} and UAS-*mCD8-GFP* or UAS-*Arpc5-IR*^{JF03147} and UAS-*P-cad.* (Right panels) Quantifications of the total area or anterior-posterior (A/P) length of adult wings in which *nub*-Gal4 drives UAS-*mCD8-GFP* (n=31) or UAS-*P-cad* and UAS-*mCherry* (n=31) or UAS-*Arp2-IR*^{JF02785} and UAS-*mCD8-GFP* (n=23) or UAS-*Arp2-IR*^{JF02785} and UAS-*P-cad* (n=25) or UAS-*Arpc3A-IR*^{JF02370} and UAS-*mCD8-GFP* (n=25) or UAS-*Arpc3A-IR*^{JF02370} and UAS-*P-cad* (n=25) or UAS-*Arpc3B-IR*^{JF02679} and UAS-*mCD8-GFP* (n=25) or UAS-*Arpc3B-IR*^{JF02679} and UAS-*P-cad* (n=25) or UAS-*Arpc4-IR*^{JF01683} and UAS-*mCD8-GFP* (n=25) or UAS-*Arpc4-IR*^{JF01683} and UAS-*P-cad* (n=25) or UAS-*Arpc5-IR*^{JF03147} and UAS-*mCD8-GFP* (n=24) or UAS-*Arpc5-IR*^{JF03147} and UAS-*P-cad* (n=25). s.p indicates square pixels. Quantification of control *nub>GFP* and *nub>mCher., P-cad* are from three biological replicates. Other quantifications are from one biological replicate. Error bars indicate SD; n.s. indicate non-significant; * indicates $P<0.05$; **** indicates $P<0.0001$**.** Statistical significance was calculated using one-way ANOVA with Tukey´s multiple comparisons tests.

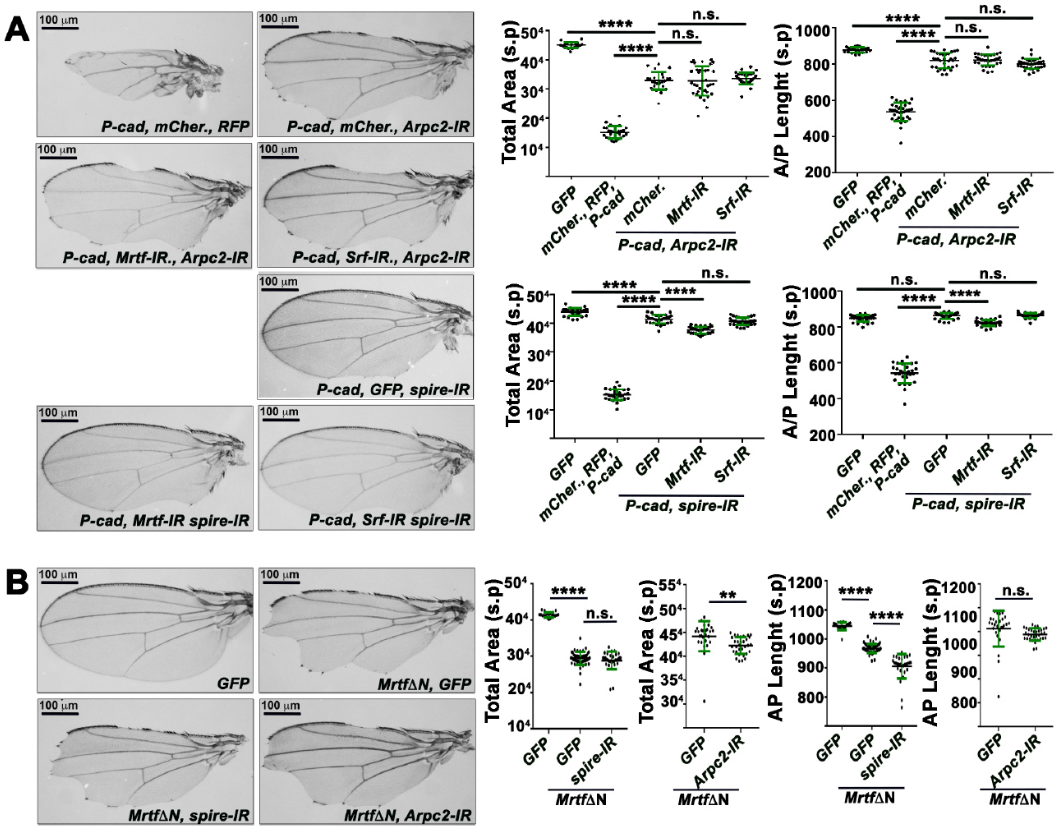

Fig. S8. ***Arpc2* and *spire* are required downstream of *P-cad* and upstream or in parallel of *MrtfΔN* to affect wing differentiation. (A)** (Left panels) Adult wings in which *nub*-Gal4 drives UAS-*P-cad*, UAS-*mCher.* and UAS-*RFP* or UAS-*P-cad*, UAS-*mCher.* and UAS-*Arpc2-IR*^{JF02845} or UAS-*P-cad*, UAS-*Mrtf-IR*^{JF02220} and UAS-*Arpc2-IR*^{JF02845} or UAS-*P-cad*, UAS-*Srf-IR*^{JF02319} and UAS-*Arpc2-IR*^{JF02845} or UAS-*P-cad*, UAS-*mCD8-GFP* and UAS-*spire-IR*^{JF03233} or UAS-*P-cad*, UAS-*Mrtf-IR*^{JF02220} and UAS-*spire-IR*^{JF03233} or UAS-*P-cad*, UAS-*Srf-IR*^{JF02319} and UAS-*spire-IR*^{JF03233}. Scale bar represents 100 µm. (Right panels) Quantifications from one biological replicate of the total area or anterior-posterior (A/P) length of adult wings in which *nub*-Gal4 drives UAS-*mCD8-GFP* (upper panels n=21; lower panels n=29) or UAS-*P-cad*, UAS-*mCher.* and UAS-*RFP* (upper panels n=26; lower panels n=30) or UAS-*P-cad*, UAS-*mCher.* and UAS-*Arpc2-IR*^{JF02845} (n=24) or UAS-*P-cad*, UAS-*Mrtf-IR*^{JF02220} and UAS-*Arpc2-IR*^{JF02845} (n=35) or UAS-*P-cad*, UAS-*Srf-IR*^{JF02319} and UAS-*Arpc2-IR*^{JF02845} (n=33) or UAS-*P-cad*, UAS-*mCD8-GFP* and UAS-*spire-IR*^{JF03233} (n=29) or UAS-*P-cad*, UAS-*Mrtf-IR*^{JF02220} and UAS-*spire-IR*^{JF03233} (n=30) or UAS-*P-cad*, UAS-*Srf-IR*^{JF02319} and UAS-*spire-IR*^{JF03233} (n=31). **(B)** (Left panels) Adult wings in which *nub*-Gal4 drives UAS-*mCD8-GFP* or UAS-*mCD8-GFP* and UAS-*MrtfΔN* or UAS-*MrtfΔN* and UAS-*spire-IR*^{JF03233} or UAS-*MrtfΔN* and UAS-*Arpc2-IR*^{JF02845}. Scale bar represents 100 µm. (Right panels) Quantifications of the total area and anterior-posterior (A/P) length of adult wings in which *nub*-Gal4 drives UAS-*mCD8-GFP* (n=18) or UAS-*mCD8-GFP* and UAS-*MrtfΔN* (n=54) or UAS-*MrtfΔN* and UAS-*spire-IR*^{JF03233} (n=31) or UAS-*MrtfΔN* and UAS-*Arpc2-IR*^{JF02845} (n=32). s.p indicates square pixels. Error bars indicate SD. n.s. indicate non-significant; ** indicates P<0.01; **** indicates P<0.0001. Statistical significance was calculated using one-way ANOVA with Tukey′s multiple comparisons tests.

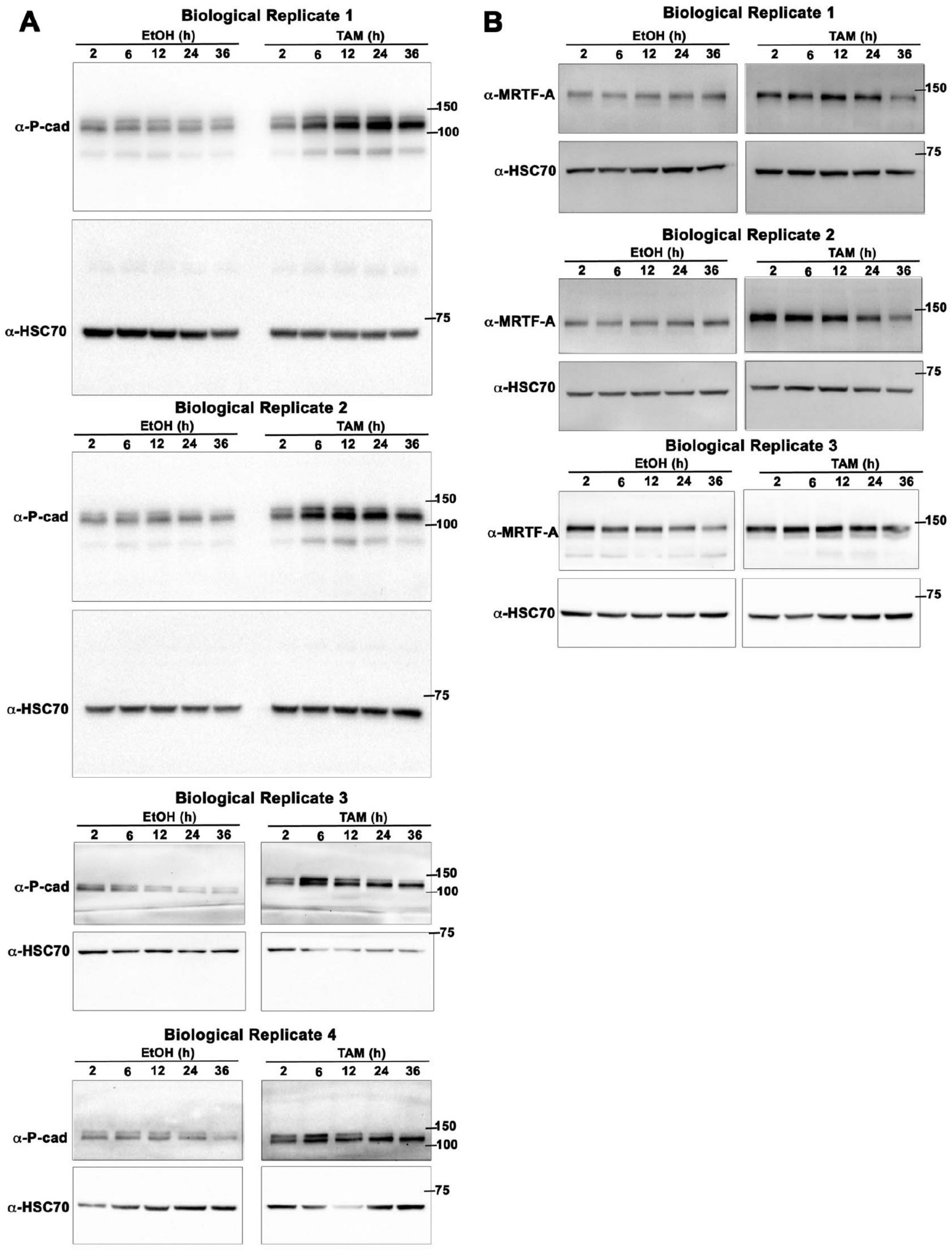

Fig. S9. P-cad and MRTF-A transiently accumulate in TAM-treated MCF10A-ER-Src cells. (A) Four biological replicates of western blots on protein extracts from MCF10A-ER-Src cells treated with EtOH or TAM for 2, 6, 12, 24 or 36 hours, blotted with anti-P-cad (upper panels) or anti-HSC70 (lower panels). **(A)** Three biological replicates of western blots on protein extracts from MCF10A-ER-Src cells treated with EtOH or TAM for 2, 6, 12, 24 or 36 hours, blotted with anti-MRTF-A (upper panels) or anti-HSC70 (lower panels).

Table S1. Primer sequences used in this study

	Primer Sequence (5' – 3')
P-cad Forward	AGTGGAGGACCCCATGAACA
P-cad Reverse	TTGGGCTTGTGGTCATTCTG
SRF Forward	TGAGTGCCACTGGCTTTGAAGAGA
SRF Reverse	AGAGGTGCTAGGTGCTGTTTGGAT
GAPDH Forward	CTCTGCTCCTCCTGTTCGAC
GAPDH Reverse	ACCAAATCCGTTGACTCCGAC
β-actin Forward	GCTCCTCCTGAGCGCAAGTA
β-actin Reverse	GATGGAGGGGCCGGACT

Disease Models & Mechanisms • Supplementary information
